# Systematic bias in malaria parasite relatedness estimation

**DOI:** 10.1093/g3journal/jkaf018

**Published:** 2025-01-30

**Authors:** Somya Mehra, Daniel E Neafsey, Michael White, Aimee R Taylor

**Affiliations:** Department of Immunology and Infectious Diseases, Harvard T.H. Chan School of Public Health, Boston, MA 02115, USA; Infectious Disease and Microbiome Program, Broad Institute of MIT and Harvard, Cambridge, MA 02142, USA; School of Mathematics and Statistics, The University of Melbourne, Parkville 3010, Australia; Department of Immunology and Infectious Diseases, Harvard T.H. Chan School of Public Health, Boston, MA 02115, USA; Infectious Disease and Microbiome Program, Broad Institute of MIT and Harvard, Cambridge, MA 02142, USA; Infectious Disease Epidemiology and Analytics G5 Unit, Institut Pasteur, Université Paris Cité, Paris 75015, France; Infectious Disease Epidemiology and Analytics G5 Unit, Institut Pasteur, Université Paris Cité, Paris 75015, France

**Keywords:** malaria, relatedness, identity by descent, hidden Markov model, independence model, bias

## Abstract

Genetic studies of *Plasmodium* parasites increasingly feature relatedness estimates. However, various aspects of malaria parasite relatedness estimation are not fully understood. For example, relatedness estimates based on whole-genome-sequence (WGS) data often exceed those based on sparser data types. Systematic bias in relatedness estimation is well documented in the literature geared towards diploid organisms, but largely unknown within the malaria community. We characterize systematic bias in malaria parasite relatedness estimation using three complementary approaches: theoretically, under a non-ancestral statistical model of pairwise relatedness; numerically, under a simulation model of ancestry; and empirically, using data on parasites sampled from Guyana and Colombia. We show that allele frequency estimates encode, locus-by-locus, relatedness averaged over the set of sampled parasites used to compute them. Plugging sample allele frequencies into models of pairwise relatedness can lead to systematic underestimation. However, systematic underestimation can be viewed as population-relatedness calibration, i.e., a way of generating measures of relative relatedness. Systematic underestimation is unavoidable when relatedness is estimated assuming independence between genetic markers. It is mitigated when relatedness is estimated using WGS data under a hidden Markov model (HMM) that exploits linkage between proximal markers. The extent of mitigation is unknowable when a HMM is fit to sparser data, but downstream analyses that use high relatedness thresholds are relatively robust regardless. In summary, practitioners can either resolve to use relative relatedness estimated under independence, or try to estimate absolute relatedness under a HMM. We propose various tools to help practitioners evaluate their situation on a case-by-case basis.

## Teaser

Patterns of relatedness between malaria parasites can yield insights on spatiotemporal scales relevant for disease control. This work addresses systematic bias in estimates of relatedness, particularly pertinent in inbred parasite populations. A combination of theory, simulation, and empirical data analysis is used to show that systematic bias can be interpreted as a means of calibration, or it can be mitigated using dense data. These findings are not entirely new in the literature that focuses on diploids, but they are largely unknown within the malaria community. Mehra *et al*. use them to construct practical guidelines for practitioners of malaria genomic epidemiology.

## Introduction

Relatedness is a genome-wide measure of identity-by-descent (IBD); i.e., identity due to common ancestry (Weir *et al.* 2006; Speed and Balding 2015). IBD is defined relative to founders, or to a previous time point, without accounting for more distant coancestry (Thompson 2013). It is a useful concept when studying malaria-causing *Plasmodium* parasites because they sexually recombine (Baton and Ranford-Cartwright 2005). Although obligate, recombination is only effective when genetically distinct parasites recombine. The probability that genetically distinct parasites recombine is context-specific: it depends on parasite diversity, on the prevalence of infected hosts, and on the within-host diversity and prevalence of polyclonal infections among infected hosts (Camponovo *et al.* 2022). As such, relatedness analyses reflect the recent, context-specific history of malaria parasites, generating epidemiologically relevant insight. For example, analyses of relatedness have been used to evaluate and inform efforts to reduce transmission (Shetty *et al.* 2019; Daniels *et al.* 2020; Morgan *et al.* 2020), to elucidate population connectivity on granular spatiotemporal scales (Omedo *et al.* 2017; Taylor *et al.* 2017, 2020; Fola *et al.* 2023; Kebede *et al.* 2023), to characterize the structure of inbred populations (de Oliveira *et al.* 2020; Carrasquilla *et al.* 2022), to resolve transmission heterogeneity (Schaffner *et al.* 2023), and to identify regions of the parasite genome subject to recent selective pressure (Henden *et al.* 2018; Amambua-Ngwa *et al.* 2019; Carrasquilla *et al.* 2022). Relatedness has further applications in clinical trials of antimalarial drugs, i.e., in the classification of *Plasmodium falciparum* reinfection and recrudescence (Plucinski *et al.* 2015) and of *Plasmodium vivax* reinfection, recrudescence, and relapse (Cowell *et al.* 2018; Taylor, Watson, *et al.* 2019).

IBD is not observable. As such, relatedness must be estimated under a statistical model. Various software exist for estimating relatedness between malaria parasites (Henden *et al.* 2018; Schaffner *et al.* 2018; Zhu *et al.* 2019; Gerlovina *et al.* 2022; Taylor 2022a). However, not all aspects of malaria parasite relatedness estimation are fully understood, especially those pertaining to systematic bias. For example, relatedness estimates based on dense whole-genome-sequence (WGS) data often exceed those based on sparse data, with striking zero-inflation of the sparse-data estimates (e.g. see Fig. S2Q of Taylor *et al.* 2017). As another example, consider the allele frequencies that are typically plugged into the models used to estimate relatedness between paired parasites. They are estimated from a sample of parasites assuming inter-parasite independence—an assumption that contradicts pairwise relatedness estimation. They are thus liable to systematically bias relatedness estimates (Rousset 2002; Wang J 2002, 2011, 2014, 2022; Yu *et al.* 2006; Bink *et al.* 2008; Kang *et al.* 2010; Weir and Goudet 2017). Weir and Goudet (2017) and others have addressed the consequent notion that relatedness is estimated relative to the average relatedness within the sample from which allele frequency estimates are computed. For diploid organisms, systematic bias is well characterized in the literature (see, for example, Anderson and Weir 2007; Bink *et al.* 2008; Wang J 2011, 2014, 2022) and alternative approaches have been proposed; for example, the KING-robust estimator, which is designed for dense data and predicated on pairwise genotype counts rather than sample allele frequencies (Manichaikul *et al.* 2010). Implications of inbreeding and “cryptic relatedness” have been addressed in the context of forensic typing (Weir 1994), and case-control studies to characterize genetic determinants of human disease (Devlin and Roeder 1999; Newman *et al.* 2001; Voight and Pritchard 2005). However, for practitioners of malaria genomic epidemiology, practical tools, and guidelines that address these biases are, to the best of our knowledge, unavailable.

Here, we characterize systematic biases in malaria parasite relatedness estimation using three complementary approaches. First, we analyze theoretically non-ancestral models of pairwise relatedness, characterizing the ramifications of the use of sample allele frequencies, shedding light on the aforementioned zero-inflation, and establishing common ground with Weir and Goudet (2017). Second, we simulate malaria parasite ancestries over successive generations of inbreeding, verifying independently our theoretical results, and elucidating systematic differences in relatedness estimates that are generated under models that assume inter-marker independence vs marker linkage due to proximity. Our numerical results help to explain differences between estimates based on sparse and dense data. Both our theoretical and numerical results assume parasites are sampled from a single population. Using data on *P. falciparum* parasites from different populations in Guyana (Vanhove *et al.* 2024) and Colombia (Carrasquilla *et al.* 2022), we illustrate how our results based on theory and simulation translate empirically. Our empirical results are context-specific. Beyond malaria genomic epidemiology, our results generalize to systems of largely haploid recombining eukaryotes (Fisher *et al.* 2002; Stauber *et al.* 2022; Wang *et al.* 2022; Huang *et al.* 2023; Sandler *et al.* 2023) or highly inbred diploid populations for which the haploid model of Leutenegger *et al.* (2003) is applicable.

## Methods

We characterize systematic biases in malaria parasite relatedness by analysing statistical models of pairwise relatedness, simulated data with known parasite ancestries, and *P. falciparum* data from Guyana and Colombia. A summary of notation used throughout our main text is provided in Table 1. A detailed description of the methods described below is available in Appendices A and B, while a glossary of terms is provided in Appendix C.

**Table 1.** Summary of terms and notation.

Terms/notation	Working definition
${\bar{\text{IBS}}}_{\ell }$	Locuswise average IBS sharing: the proportion of pairs in a sample (including self–self comparisons) that are IBS at a given marker ℓ
${\bar{\text{IBD}}}_{\ell }$	Locuswise average IBD sharing: the proportion of pairs in a sample (including self–self comparisons) that are IBD at a given marker ℓ
$\text{mean}({\bar{\text{IBD}}}_{\ell })$	Locuswise average IBD sharing averaged over all markers ℓ=1,…
*r*	Relatedness parameter governing the marginal probability of IBD at each marker under non-ancestral models of pairwise relatedness
$\hat{r}$	Maximum likelihood estimate of the parameter *r*; an estimate of pairwise relatedness
realized relatedness	Fraction of polymorphic markers across the genome that are IBD for a parasite pair

### Statistical models of pairwise relatedness

We derive theoretical results and estimate pairwise relatedness using models that couple a non-ancestral model of latent IBD and non-IBD (nIBD) states describing a pair of parasites along a sequence of marker loci and a locuswise observation model.

The latent state model either assumes independence between markers or accounts for marker linkage under the intuition that IBD segments are fragmented at randomly-distributed recombination breakpoints over successive generations, with a genome-wide-constant recombination rate (Stam 1980; Leutenegger *et al.* 2003; Taylor, Jacob, *et al.* 2019). Of key interest is the pairwise relatedness parameter *r*, which describes the marginal probability that a given marker locus is IBD for a parasite pair, i.e., if we consider a single locus in isolation under the model then, P(IBD)=r.

The locuswise observation model provides an explicit link between latent and observable states. Observable states are either pairs of alleles or descriptives of identity-by-state (IBS); i.e., IBS for a pair of identical alleles and non-IBS (nIBS) otherwise. For IBS descriptives, the observational model takes the form $P_{\mathrm{obs}}(\text{IBS}\;|\;\text{IBD})=1$ in the absence of genotyping error (since IBD necessarily implies IBS) while $P_{\mathrm{obs}}(\text{IBS}\;|\;\text{nIBD})$ must be defined appropriately.

Under the coupled model of relatedness, the marginal likelihood of IBS at a given marker can be written


(1) $$P(\text{IBS}\;|\;r)=r+P_{\mathrm{obs}}(\text{IBS}\;|\;\text{nIBD})\cdot (1-r).$$

Here, we focus on maximum likelihood estimates $\hat{r}$ as an estimate of relatedness between a pair of malaria parasites. We do not address the estimation or identification of IBD segments.

It is standard practice to estimate allele frequencies from data on a set of sampled parasites (either before or after removing replicates of apparent clones) and plug them into observation models. We refer to these frequency estimates as sample allele frequencies and to the observation models into which they are plugged as standard practice observation models. Other terms we use include (n)IBD to refer to IBD and nIBD collectively, (n)IBS to refer to IBS and nIBS collectively, hidden Markov model (HMM) to refer to models of relatedness that allow for linkage between proximal markers, and independence model to refer to models of relatedness that assume marker loci are independent. We use the word model to refer to models of relatedness, the models within the models of relatedness (i.e., (n)IBD models and (n)IBD-to-observation models) and the models within the (n)IBD models and (n)IBD-to-observation models (e.g., IBD-to-allele model). We use the term fraction to indicate pairwise averages over loci across the genome, and proportion to indicate locuswise averages over parasites within a population.

Here, we are primarily concerned with bias stemming from standard practice observation models. As a theoretical comparator, we adopt a corrected model of (n)IBS descriptives with conceptual parallels to Weir and Goudet (2017). The corrected model is not available practically, but illustrates theoretically the partial encoding of population relatedness in the sums of squares of sample allele frequencies. Further details of the observation models are provided in the results section. For clarity, genotyping error is not modeled and theoretical derivations are restricted to the independence model of relatedness.

### Simulation model of parasite ancestries

While our theoretical results are derived under a non-ancestral pairwise framework, we perform numerical analyses under an ancestrally-informed simulation of a single parasite population. Although our simulation framework is highly simplified and does not fully recapitulate epidemiological reality, it enables independent verification of our theoretical results and allows us to examine the consequences of marker linkage.

A detailed description of the simulation model is provided in Appendix B. Under our simulation framework, IBD is defined relative to founders that pre-date parasites in generation zero. Generation zero is a key time point at which we fix the parasite population size and thus initiate inbreeding (e.g., the start of some control strategy), and the time point against which background and recent relatedness is defined: IBD present in generation zero is low-level and spread maximally across parasite genotypes; recent IBD that post-dates generation zero can range from low to high level and is concentrated on subsets of parasite genotypes. The main assumptions of the simulation model are as follows:

- Sufficient temporal separation between “generation zero” and a bygone outbred founder population to break down marker linkage, whereby the ancestry of each individual in generation zero is sampled uniformly over the set of all possible founder mosaics; this construction distributes ancient low-level background relatedness broadly across the generation zero population.
- Discrete, non-overlapping generations of inbreeding from generation zero onwards, modeled using a transitive relationship graph with sibling/clonal/stranger edges as per Taylor, Watson, *et al.* 2019; relationship graphs are obtained by amalgamating uniformly sampled subgraphs of a fixed size (Taylor 2022b), biasing the distribution towards small, balanced clusters of siblings and clones.
- Stochastic drift arising from the random crossing of parental genotypes from generation (k−1) to yield filial genotypes in generation *k*, with an augmented probability of selfing and sibling–sibling crosses serving as a proxy for monoclonal mosquito infection and serial cotransmission (Wong *et al.* 2018).
- The absence of immigration, mutation, selection, and population structure under a small, fixed parasite population size.
- A genome-wide-constant recombination rate, with markers treated as nominal point polymorphisms.

When we analyze simulated data, the ground truth against which we characterize systematic bias in pairwise relatedness estimates is the fraction of polymorphic marker loci that are IBD for a given parasite pair; otherwise known as realized relatedness (Speed and Balding 2015). Akin to the Wright–Fischer model, in which fixation necessarily occurs, the number of polymorphic markers tends to decrease over successive generations of inbreeding under our simulation model. Because they are predicated on data from polymorphic markers only, pairwise relatedness estimates are liable to increased variability as the set of informative markers is thinned over successive generations. For comparability, we compute realized relatedness over polymorphic markers. There is a conceptual distinction between realized relatedness and the relatedness parameter *r* of the non-ancestral pairwise model: given a finite number of markers, a fixed value of *r* gives rise to a *distribution* of realized relatedness (Speed and Balding 2015; Taylor, Jacob, *et al.* 2019). However, since we simulate recombination from first principles, our simulation framework does not yield a direct analogue for the non-ancestral relatedness parameter *r*.

### Case study of *P. falciparum* data from Guyana and Colombia

Using a case study, we show our theoretical and numerical results are practically relevant. Specifically, we analyze a monoclonal subset of high-quality, whole-genome-sequenced *P. falciparum* isolates from passively sampled symptomatic patients in Guyana between 2016 and 2020 (Vanhove *et al.* 2024) and Colombia between 1993 and 2017 (Carrasquilla *et al.* 2022). Variants were called in accordance with best practices stipulated by GATK and the MalariaGEN Pf3k consortium (Carrasquilla *et al.* 2022; Vanhove *et al.* 2024). We restrict our attention to biallelic SNPs that are in core regions of the nuclear genome (Miles *et al.* 2016) and are polymorphic among the isolates, masking any variant calls that are heteroallelic or have read support below five (based on the DP tag). We remove SNPs with missingness >30% across isolates and isolates with missingness >30% across SNPs to yield a WGS dataset comprising n=306 isolates (n=278 from Guyana, n=28 from Colombia) and n=30,694 SNPs. Because our theoretical and numerical results assume parasites are sampled from a single population, results in the main text are restricted to the n=278 isolates from Guyana, among which n=16,115 SNPs are polymorphic. Sparse datasets are generated by downsampling SNPs uniformly at random without replacement.

### Data analyses

All data analyses are done in R (R Core Team 2021). Relatedness estimates based on allelic states are generated using the R package paneljudge (Taylor 2022a), which implements the HMM with allelic observations and its independent counterpart, and employs a maximum likelihood estimation scheme. The model of independent (n)IBD states with (n)IBS observations has been custom coded in R. We do not estimate relatedness using a HMM with (n)IBS observations. All code and a minimum analysis dataset for the empirical case study is available in an accompanying GitHub repository (Mehra *et al.* 2024): https://doi.org/10.5281/zenodo.14176553

## Results

A detailed description of the results below can be found in Appendix A, which starts with an overview of the support (theoretical, numerical, and/or empirical) per result (Table A1). Our theoretical and numerical results are conditioned on a single parasite population; an analysis of empirical data in the presence of population structure is deferred to Appendix A4.

### Theoretical results

#### Standard (n)IBD-to-observation models are twice misspecified

In standard practice, (n)IBD-to-observation models are informed by sample allele frequencies (Wang J 2014; Taylor *et al.* 2017, 2020; Henden *et al.* 2018; Taylor, Jacob, *et al.* 2019; Zhu *et al.* 2019). Taylor, Jacob, *et al.* (2019) allude to potential misspecification arising from this construction: while relatedness estimation seeks to estimate dependence between individuals, the sample allele frequencies, and thus any observation models into which they are plugged, are constructed under the implicit assumption of independence between individuals. Implications for diploid organisms have been addressed extensively by Wang J (2014) and others. Weir and Goudet (2017) have articulated the notion that relatedness is consequently estimated relative to the average relatedness within the sample.

Here, we argue that standard (n)IBD-to-observation models are twice misspecified:

1. The standard IBD-to-allele model, whereby the probability of an IBD pair exhibiting allele *q* is given by the sample frequency of allele *q*, is implicitly predicated on the independence of allelic and IBD states which may not hold in reality (Equation (A10)), particularly in the presence of selection.
2. The standard nIBD-to-IBS model, under which the probability of an IBS observation given a nIBD latent state is equal to the proportion of IBS pairs in the set of sampled parasites (calculated by taking the sums of squares of sample allele frequencies), is inflated by the encoding of average locuswise relatedness (Equation (A12)). Weir and Goudet (2017) similarly exploit IBS descriptives rather than allelic states for conceptual clarity.

Removing clonal replicates will not circumvent IBD-to-allele misspecification although it seems reasonable to expect it might mitigate it; otherwise, consequences of the IBD-to-allele misspecification are case-specific and beyond the scope of our current study. For the remainder of this study, we focus on nIBD-to-IBS misspecification because it is more pervasive and has systematic consequences.

An illustration of nIBD-to-IBS misspecification is shown in Box 1, echoing the work of Weir and Goudet (2017). We can summarize this form of misspecification mathematically as follows. Denote by $\bar{\text{IBS}}$ and $\bar{\text{IBD}}$ the proportion of pairs of sampled parasites (including self–self comparisons) that are IBS and IBD, respectively, at a given locus (Table 1, but with locus identifiers dropped for notational convenience). Under the standard nIBD-to-IBS model, we set


(2) $$P_{\mathrm{standard}}(\text{IBS}\;|\;\text{nIBD})=\bar{\text{IBS}}.$$

A proportion of IBS sharing in $\bar{\text{IBS}}$, however, is attributable to IBD. To adjust for locuswise relateness in the set of sampled parasites, we would need to average IBS sharing over nIBD pairs only, that is,


(3) $$P_{\mathrm{corrected}}(\text{IBS}\;|\;\text{nIBD})=\frac{\bar{\text{IBS}}-\bar{\text{IBD}}}{1-\bar{\text{IBD}}}.$$

The correction (Equation (3)) cannot be implemented in practice because (n)IBD states are unobservable. Nonetheless, it provides a theoretical basis for understanding the misspecification of the standard nIBD-to-IBS model. To see how relatedness structure, in the form of locuswise average relatedness $\bar{\text{IBD}}$, is implicitly embedded in the standard nIBD-to-IBS model, we rearrange Equation (3) to yield


(4) $$\begin{array}{rl} P_{\mathrm{standard}}(\text{IBS}\;|\;\text{nIBD}) & =\bar{\text{IBD}}+(1-\bar{\text{IBD}})\cdot P_{\mathrm{corrected}}(\text{IBS}\;|\;\text{nIBD}),\\ & >P_{\mathrm{corrected}}(\text{IBS}\;|\;\text{nIBD}), \end{array}$$

where the strict inequality is just a technicality. Since we have included self–self comparisons (i.e. comparisons with replacement) for consistency with standard nIBD-to-allele models (Henden *et al.* 2018; Schaffner *et al.* 2018), $\bar{\text{IBD}}=1/n>0$ for an outbred sample of size *n*. The finite sample adjustment of Purcell *et al.* (2007), which considers pairwise comparisons without replacement, yields $\bar{\text{IBD}}=0$ for an outbred sample. However, under either construction, the form of the theoretical correction to the nIBD-to-IBS model is identical.

Box 1: **Illustrating misspecification of the standard nIBD-to-IBS model**

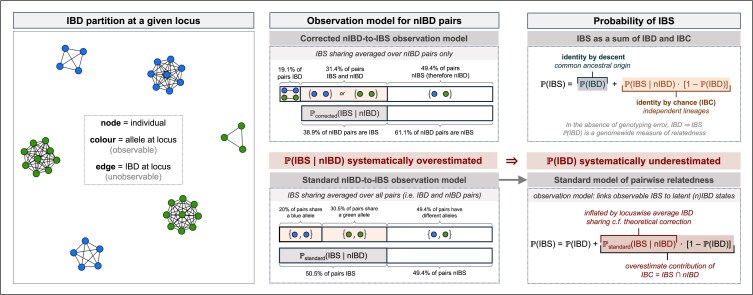

**Standard nIBD-to-IBS model**

At a given locus, b=17 blue nodes and g=21 green nodes are observed. The proportion of pairs that are IBS, which we denote $\bar{\text{IBS}}$, can be written as the sum of squares of the blue b/(b+g) and green g/(b+g) allele frequencies:

$$\bar{\text{IBS}}=(b^{2}+g^{2})/(b+g{)}^{2}=0.506.$$

Under the standard nIBD-to-IBS model the probability of an IBS pair given an nIBD pair is equal to the overall proportion of pairs that are IBS:

$$P_{\mathrm{standard}}(\text{IBS}\;|\;\text{nIBD})=\bar{\text{IBS}}=0.506.$$

**Corrected nIBD-to-IBS model**

There are three green clusters in the IBD partition, of size $c_{1}=9$, $c_{2}=9$, and $c_{3}=3$. Out of a total of $(b+g{)}^{2}=1,444$ possible pairwise comparisons, this means that

$$(c_{1}^{2}+c_{2}^{2}+c_{3}^{2})/(b+g{)}^{2}=0.118\;\text{of pairs are IBD}\;and\;\text{share green alleles}$$

Likewise, since there are three blue clusters of size $s_{1}=8$, $s_{2}=5$, and $s_{3}=4$

$$(s_{1}^{2}+s_{2}^{2}+s_{3}^{2})/(b+g{)}^{2}=0.073\;\text{of pairs are IBD}\;and\;\text{share blue alleles}$$

The proportion of pairs which are IBD is then

$$\bar{\text{IBD}}=0.118+0.073=0.191,$$

while the proportion of pairs that are IBS and nIBD is

$$\bar{\text{IBS}}-\bar{\text{IBD}}=0.506-0.191=0.314.$$

To construct a model of IBS specifically for nIBD pairs, we would ideally focus on IBS sharing in the proportion of $(1-\bar{\text{IBD}})=0.809$ pairs that are nIBD, that is,

$$P_{\mathrm{corrected}}(\text{IBS}\;|\;\text{nIBD})=\frac{\bar{\text{IBS}}-\bar{\text{IBD}}}{1-\bar{\text{IBD}}}=0.389.$$

**Misspecification of the standard nIBD-to-IBS model**

In failing to adjust for IBD sharing under the standard nIBD-to-IBS model, we have overestimated the probability of IBS sharing for nIBD pairs:

$$P_{\mathrm{standard}}(\text{IBS}\;|\;\text{nIBD})=0.506>0.389=P_{\mathrm{corrected}}(\text{IBS}\;|\;\text{nIBD}).$$

In summary, the probability of IBS sharing for nIBD pairs is systematically overestimated under the standard nIBD-to-IBS model, with a particularly pronounced effect in inbred populations with large $\bar{\text{IBD}}$.

#### Misspecification: relative and zero-valued relatedness estimates

Here, we examine systematic bias in maximum likelihood estimates ${\hat{r}}_{\mathrm{standard}}$ generated under the standard nIBD-to-IBS model (Equation (2)), relative to hypothetical estimates ${\hat{r}}_{\mathrm{corrected}}$ generated under the corrected nIBD-to-IBS model (Equation (3)). For conceptual clarity, we enforce the assumption of marker independence (consequences of marker linkage are explored in our numerical analyses).

We can intuit that misspecification of the standard nIBD-to-IBS model will lead to systematic underestimation of pairwise relatedness, that is, ${\hat{r}}_{\mathrm{standard}}<{\hat{r}}_{\mathrm{corrected}}$ (Box 1). Two additional consequences for which we have theoretical support are as follows:

1. The standard pairwise relatedness parameter $r_{\mathrm{standard}}$ can be reinterpreted as a relative measure of deviation from population-averaged locuswise relatedness (Appendix A.2.2.1). The marginal likelihood of IBS sharing at a given marker ℓ under the standard model with relatedness parameter $r_{\mathrm{standard}}$ is equivalent to the likelihood under the corrected model with relatedness parameter $r_{\mathrm{corrected}}=r_{\mathrm{standard}}+{\bar{\text{IBD}}}_{\ell }[1-r_{\mathrm{standard}}]$, where ${\bar{\text{IBD}}}_{\ell }$ is the proportion of sampled parasites that are IBD at locus ℓ, that is,
  $$\begin{array}{rl} & P_{\mathrm{standard}}({\text{IBS}}_{\ell }\;|\;r_{\mathrm{standard}})\\ & \;=P_{\mathrm{corrected}}({\text{IBS}}_{\ell }\;|\;r_{\mathrm{corrected}}=(r_{\mathrm{standard}}+{\bar{\text{IBD}}}_{\ell }[1-r_{\mathrm{standard}}])). \end{array}$$
  In practice, the average locuswise relatedness ${\bar{\text{IBD}}}_{\ell }$ will vary across loci ℓ. In a hypothetical population with identical average locuswise relatedness over all loci, ${\bar{\text{IBD}}}_{\ell }={\bar{\text{IBD}}}_{\mathrm{constant}}$, we obtain the functional relationship
  (5) $$r_{\mathrm{standard}}=\frac{r_{\mathrm{corrected}}-{\bar{\text{IBD}}}_{\mathrm{constant}}}{1-{\bar{\text{IBD}}}_{\mathrm{constant}}}.$$
  This interpretation of relative relatedness echoes the work of Weir and Goudet (2017).
2. Relatedness estimates can be stratified by average sample relatedness: zero for parasite pairs with relatedness below the sample average, positive otherwise (Appendix A.2.2.2). To understand why this is the case, using Equations (1) and (4), we observe that
  (6) $$\begin{array}{rl} & P_{\mathrm{standard}}({\text{IBS}}_{\ell }\;|\;r_{\mathrm{standard}}=0)\\ & \;={\bar{\text{IBD}}}_{\ell }+(1-{\bar{\text{IBD}}}_{\ell })P_{\mathrm{corrected}}({\text{IBS}}_{\ell }\;|\;{\text{nIBD}}_{\ell })\\ & \;=P_{\mathrm{corrected}}({\text{IBS}}_{\ell }\;|\;r_{\mathrm{corrected}}={\bar{\text{IBD}}}_{\ell }). \end{array}$$
  That is, plugging $r_{\mathrm{standard}}=0$ into the standard model likelihood of IBS at a given marker ℓ is equivalent to plugging in the average locuswise relatedness $r_{\mathrm{corrected}}={\bar{\text{IBD}}}_{\ell }$ into the corrected model likelihood. In other words, population-averaged locuswise relatedness is implicitly encoded in the standard model even in the case $r_{\mathrm{standard}}=0$, and parasite pairs with less IBS sharing than predicted under population-averaged relatedness (given explicitly by the threshold (A25)) are assigned zero estimates ${\hat{r}}_{\mathrm{standard}}=0$. Similar observations have been made previously by Hall *et al.* (2012), Weir and Goudet (2017) and others. Moment estimators, which permit negative estimates unlike the maximum likelihood estimators, may be more informative for parasite pairs with relatedness below the sample average (Hall *et al.* 2012).

**Fig. 1.**
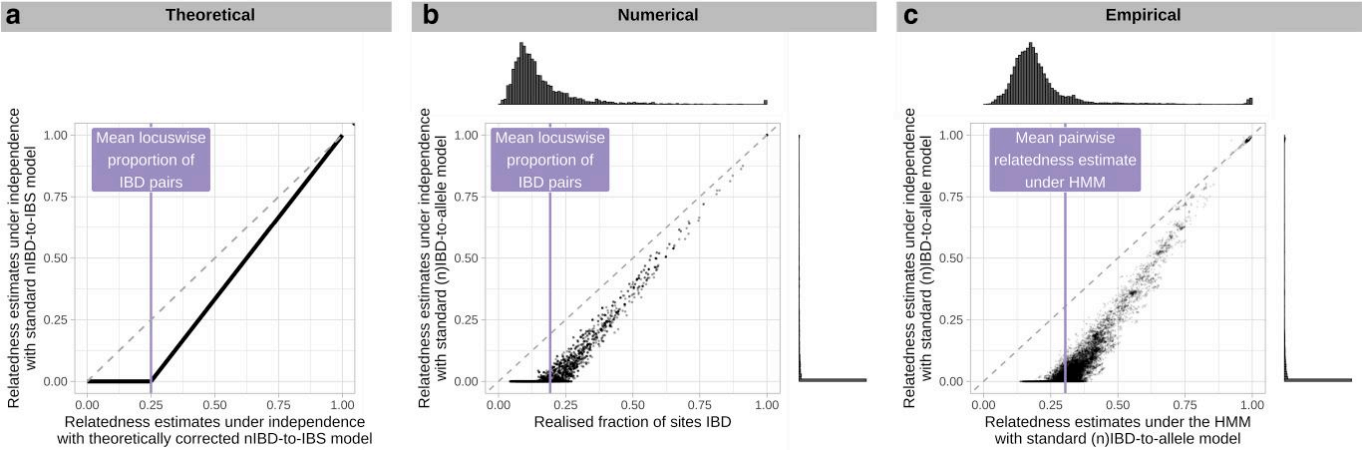
Characterization of systematic bias in pairwise relatedness under (n)IBD independence. We characterize bias against three comparators: a) ${\hat{r}}_{\mathrm{corrected}}$ estimates computed theoretically; b) realized relatedness computed using simulated data; and c) ${\hat{r}}_{\mathrm{standard}}$ estimates computed using the HMM fit to WGS *P. falciparum* data from Guyana. In cases (a) and (b), we recover an elbow-like characteristic with change point near the mean locuswise proportion of IBD pairs $\text{mean}({\bar{\text{IBD}}}_{\ell })$.

Accounting for variability in ${\bar{\text{IBD}}}_{\ell }$ across loci ℓ, we predict estimates ${\hat{r}}_{\mathrm{standard}}$ against the theoretical comparator ${\hat{r}}_{\mathrm{corrected}}$ to exhibit a fuzzy elbow-like characteristic, with a change point in the vicinity of the mean locuswise average IBD sharing, $\text{mean}({\bar{\text{IBD}}}_{\ell })$ (Fig. 1a). If the distribution of ${\hat{r}}_{\mathrm{corrected}}$ is unimodal and positively skewed, we expect pronounced zero inflation in estimates of $r_{\mathrm{standard}}$ under the standard nIBD-to-IBS model.

### Numerical results

#### Theoretical validation

**Fig. 2.**
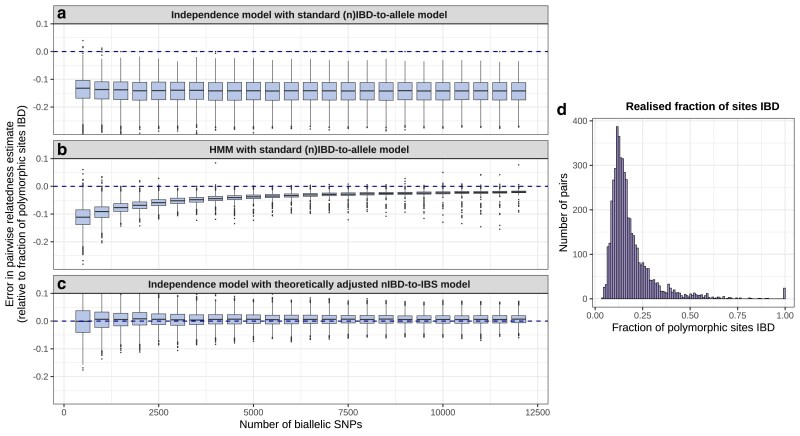
Summary of pairwise relatedness for simulated data after 10 generations of inbreeding. Systematic bias is shown as a function of marker density, with the fraction of (polymorphic) marker loci IBD taken as the ground truth, for a) ${\hat{r}}_{\mathrm{standard}}$ under (n)IBD independence and the standard (n)IBD-to-allele model; b) ${\hat{r}}_{\mathrm{standard}}$ computed using the HMM and the standard (n)IBD-to-allele model; c) ${\hat{r}}_{\mathrm{corrected}}$ under (n)IBD independence and the corrected nIBD-to-IBS model. d) Histogram of realized relatedness (fraction of polymorphic sites that are IBD for simulated parasites).

Estimates generated under the independence model of relatedness using simulated data support theoretical results as follows. In line with standard nIBD-to-IBS model misspecification, systematic bias in estimates of $r_{\mathrm{standard}}$ increases as $\text{mean}({\bar{\text{IBD}}}_{\ell })$ increases (Appendix A.3.1). In line with the proposed nIBD-to-IBS model correction, ${\hat{r}}_{\mathrm{corrected}}$ are largely unbiased (Appendix A.3.2, Fig. 2a vs 2c). Bias-mitigation under (n)IBD independence supports the notion that systematic bias of ${\hat{r}}_{\mathrm{standard}}$ can be attributed to the partial encoding of sample relatedness in standard observation models. In line with expected zero-valued estimates, ${\hat{r}}_{\mathrm{standard}}=0$ for simulated pairs that exhibit a smaller IBS fraction than that which is expected for $r_{\mathrm{standard}}=0$ under the standard nIBD-to-observation model (Appendix A.3.4). In line with Fig. 1a, plots of ${\hat{r}}_{\mathrm{standard}}$ against realized relatedness yield an elbow-like characteristic, branching approximately at $\text{mean}({\bar{\text{IBD}}}_{\ell })$ (Fig. 1b).

#### Exploiting marker linkage can mitigate bias

While systematic underestimation given (n)IBD independence persists irrespective of marker density (Fig. 2a), it is mitigated as a function of marker density when data are analyzed under the HMM (Fig. 2b). We attribute this trend to the exploitation of increasingly detailed linkage information under the HMM, reducing the reliance of $r_{\mathrm{standard}}$ estimation on the standard observation model, which is misspecified. We thus propose a dense data diagnostic that leverages increased precision under the HMM (Appendix A.3.5): for dense data, comparison of ${\hat{r}}_{\mathrm{standard}}$ estimates under (n)IBD independence vs the HMM is expected to yield an elbow-like characteristic (analogous to Fig. 1b), which can be used to ascertain both the severity of underestimation and approximate $\text{mean}({\bar{\text{IBD}}}_{\ell })$.

For sparse data that do not encode linkage information, analyses of simulated data under the independence model of relatedness confirm that bias-mitigation would be possible if model-adjustment were available, and that model-adjustment would be sufficient (Fig. 2c).

#### Relative vs absolute relatedness

**Fig. 3.**
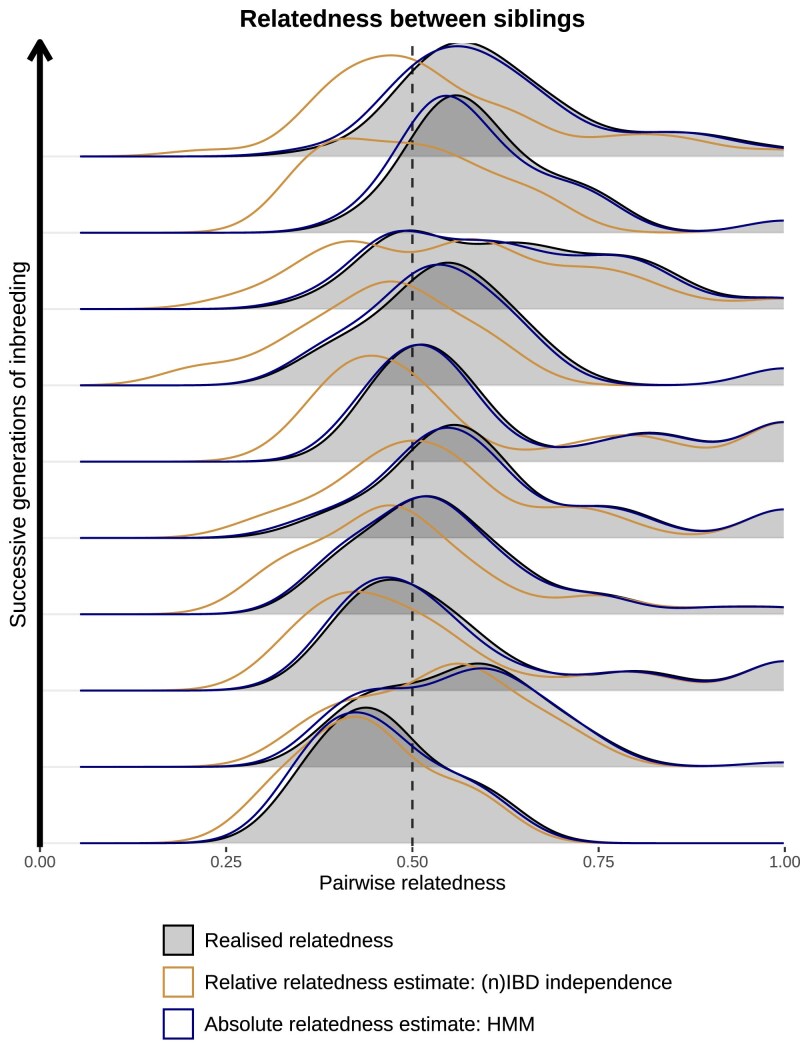
Summary of pairwise relatedness for simulated siblings across successive generations of inbreeding. We compare realized relatedness (i.e., the fraction of (polymorphic) sites that are IBD for a given parasite pair) against ${\hat{r}}_{\mathrm{standard}}$ predicated on the standard (n)IBD-to-allele model with (n)IBD independence (relative relatedness estimates) vs the HMM (absolute relatedness estimates).

Across successive generations of inbreeding, realized relatedness between siblings (gray shading, Fig. 3) is systematically enriched above 0.5. For dense data, we can interpret ${\hat{r}}_{\mathrm{standard}}$ under the HMM (navy blue, Fig. 3) as a measure of absolute relatedness which recapitulates this enrichment. In contrast, ${\hat{r}}_{\mathrm{standard}}$ for siblings under (n)IBD independence (orange, Fig. 3) is generally centred around 0.5, supporting interpretation as a measure of relative relatedness. This points towards two choices for practitioners: the analysis of absolute relatedness under a HMM, which requires sufficiently dense genotypic data; or the use of relative relatedness under (n)IBD independence, which warrants careful interpretation in cross-population comparisons (the degree of bias may vary across transmission settings, but relative relatedness may have utility, for instance, as a relationship proxy).

### Empirical results

#### Case study: inbred parasite population from Guyana

To illustrate the practical implications of our findings, we analyze WGS data from n=278 high-quality *P. falciparum* isolates (deemed to be monoclonal) sampled from patients in Guyana in 2016–2020 (Vanhove *et al.* 2024).

Based on our numerical results (specifically, Fig. 2b), estimates of relatedness generated under the HMM using WGS data are expected to exhibit relatively little bias and therefore serve as a pragmatic gold-standard. To gauge the severity of systematic underestimation due to standard observation model misspecification, we draw on the proposed dense data diagnostic: a comparative plot of dense data ${\hat{r}}_{\mathrm{standard}}$ estimates generated under the independence model and the HMM (Fig. 1c). The position of the change point suggests that the mean locuswise proportion of IBD pairs is in the vicinity of $\text{mean}({\bar{\text{IBD}}}_{\ell })\approx 30\%$. Across parasite pairs, the average estimate ${\hat{r}}_{\mathrm{standard}}$ under the HMM using WGS data is 30.4% (vertical line, Fig. 1c).

**Fig. 4.**
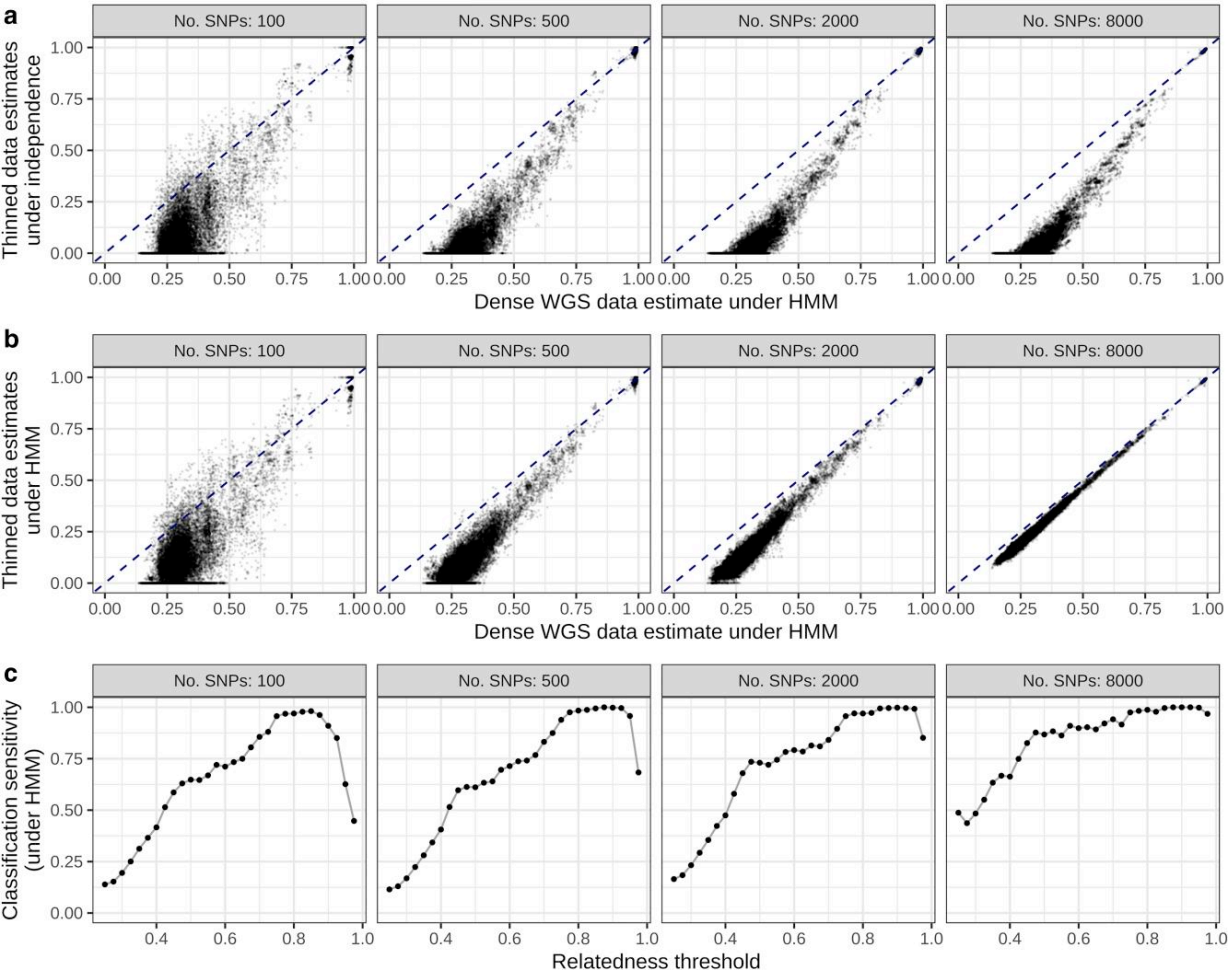
Consequences of marker sparsity on pairwise relatedness estimates for 278 *P. falciparum* isolates from Guyana. Thinned data are generated by downsampling SNPs in the dense data (complete WGS data) uniformly at random without replacement. a) Relatedness estimated under (n)IBD independence with thinned data vs the HMM with dense data. b) Relatedness estimated under the HMM with thinned data vs dense data. c) Related-pair classification sensitivity of relatedness estimated under the HMM fit to thinned data. For each threshold, we record a true positive (TP) if estimates generated under the HMM for both thinned and dense data exceed the threshold; and a false negative (FN) if only the HMM estimate for dense data exceeds the threshold; sensitivity is given by the ratio TP/(TP+FN). All estimates are generated using the standard (n)IBD-to-allele model.

In addition to systematic bias stemming from model misspecification, uncertainty due to marker sparsity may become significant for sparse data (Taylor *et al.* 2020). We expect the point at which uncertainty/variance obfuscates the elbow-like characteristic to be dependent on the average relatedness within the set of sampled parasites. A sparse-dense data diagnostic, comprising comparative plots of estimates generated under (n)IBD independence using sparse data vs estimates generated under the HMM using dense data, can be used to elucidate this trade-off because under (n)IBD independence, increasing marker density does not mitigate systematic bias but does reduce uncertainty in pairwise relatedness estimates. For n=278  *P. falciparum* isolates from Guyana, Fig. 4a suggests bias due to elevated population relatedness dominates uncertainty due to marker sparsity: even at low marker densities, systematic underestimation is apparent.

A possible strategy for offsetting systematic bias is to exploit linkage information under the HMM regardless of marker density. However, systematic underestimation is only partially mitigated when the HMM is fit to sparse data (Fig. 4b). Unlike estimates under the independence model—which can be interpreted as relative measures—sparse data HMM estimates do not have a clear interpretation.

The error structure in sparse data HMM estimates is heteroskedastic: underestimation is most pronounced for parasite pairs with low relatedness. Genomic epidemiology applications typically focus on highly related parasite pairs, often using a threshold-based approach (Taylor *et al.* 2017; Henden *et al.* 2018; Miotto *et al.* 2020; Fola *et al.* 2023; Carrasquilla *et al.* 2022; Harrison *et al.* 2023). Since relatedness is systematically underestimated using sparse data, false positives (pairs with low levels of relatedness that appear highly related) are relatively rare. The sensitivity of sparse data HMM estimation in identifying pairs with relatedness above a threshold is shown in Fig. 4c. When thresholds are high, sparse data HMM estimates suffice; sensitive classification for low thresholds, however, requires higher resolution data (Harrison *et al.* 2023). We are reluctant to posit marker density thresholds necessary to sensitively identify highly related parasite pairs in generality, because the degree of linkage structure within a set of sampled parasites depends on the distribution of shared IBD segment lengths—which, in turn, is driven by demographic processes that we neither fully understand, nor control. Downsampling WGS data provides insight on a case-by-case basis.

#### Population structure

**Fig. A12.**
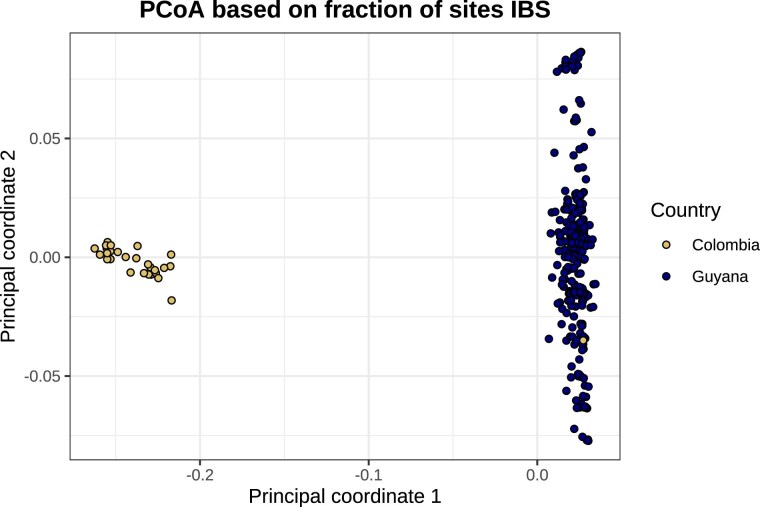
Principal coordinates analysis, with a distance matrix based on pairwise fractions of IBS markers. PCoA has been performed using the R function stats::cmdscale (R Core Team 2021).

Since our theoretical and numerical results assume a single parasite population, we additionally analyze jointly the n=278 isolates from Guyana (Vanhove *et al.* 2024) and n=28 isolates from Colombia (Carrasquilla *et al.* 2022). Principal coordinates analysis (PCoA) of pairwise fractions of IBS markers yields two distinct clusters, stratified by country (Fig. A.12). A multimodal distribution of IBS fractions, and systematic differences between relatedness estimates under the standard nIBD-to-IBS vs (n)IBD-to-allele model corroborate the presence of population structure (Appendix A.4.1), and aid in explaining deviations from our theoretical and numerical results because they are conceptually aligned with the present framework.

**Fig. A15.**
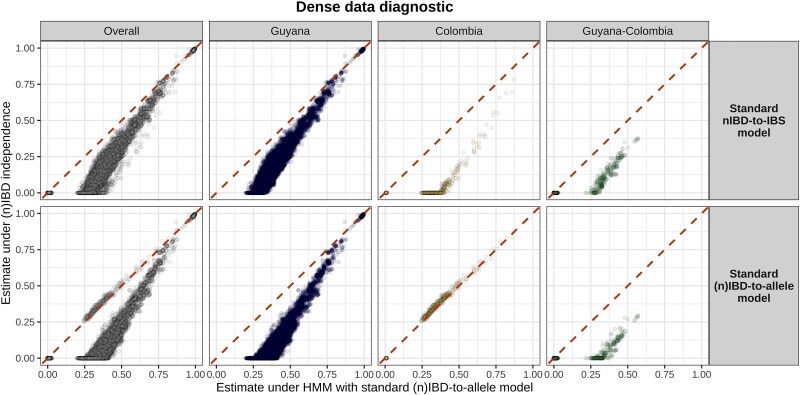
Dense data diagnostic, comparing relatedness estimates under the HMM and (n)IBD independence, using the standard nIBD-to-IBS or (n)IBD-to-allele model. We stratify comparisons within and between countries.

**Fig. A16.**
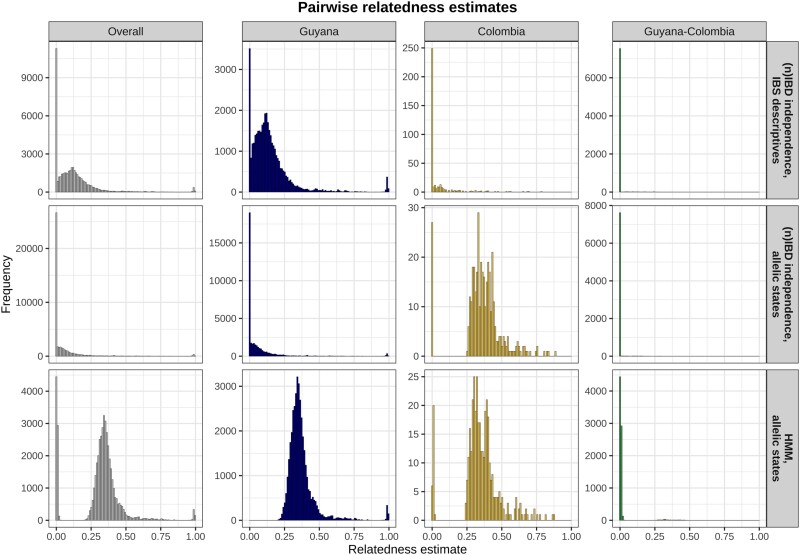
Pairwise relatedness estimated under (n)IBD independence with the standard nIBD-to-IBS model; (n)IBD independence with the standard (n)IBD-to-allele model; and the HMM with the standard (n)IBD-to-allele model. We stratify comparisons within and between countries.

In this setting, the dense data diagnostic is characterized by multiple elbows, corresponding to different within- and between-population comparisons (Fig. A.15), and the severity of zero-inflation under (n)IBD independence varies for the major/minor subpopulation depending on the use of allelic states or IBS descriptives (Fig. A.16). Given sample allele frequencies constitute a weighted average across subpopulations, the interpretation of zero inflation and branch points in the dense data diagnostic is unclear; an observation model predicated on subpopulation-stratified allele frequencies (Schaffner *et al.* 2018), may yield more interpretable results. Further discussion is provided in Appendix A.4.

## Discussion

### Summary and interpretation of results

We characterize systematic bias in malaria parasite relatedness estimation, providing theoretical, numerical and/or empirical support for our results (Table A1). In the context of diploid organisms, biases in relatedness estimation are well established (Anderson and Weir 2007; Bink *et al.* 2008; Wang J 2014), and estimators that are more robust to inbreeding are available (Manichaikul *et al.* 2010; Wang J 2011, 2022). In the context of malaria parasites, which are haploid and can self, biases in relatedness estimation are not broadly recognized. Our results are not limited to malaria parasites, and extend to any system concerned with pairwise relatedness of predominately haploid recombining eukaryotes (e.g. *Cryptosporidium hominis* (Huang *et al.* 2023) and *Cryptosporidium parvum* (Wang *et al.* 2022), leading causes of human and zoonotic cryptosporidiosis respectively; *Coccidioides* species which give rise to human coccidioidomycosis (Fisher *et al.* 2002); *Cryphonectria parasitica*, the pathogenic agent responsible for Chestnut blight (Stauber *et al.* 2022) and *Marchantia polymorpha*, a model species of liverwort (Sandler *et al.* 2023)) or highly inbred populations of diploid organisms for which pairwise relatedness can be interrogated using a haploid model (Leutenegger *et al.* 2003).

Relatedness estimates with relatively low root mean squared error can be generated using moderate marker counts under a standard model of pairwise relatedness when it is well specified (Taylor, Jacob, *et al.* 2019). Misspecification of standard (n)IBD-to-observation models, which are predicated on sample allele frequencies, constitutes our conceptual starting point (Rousset 2002; Wang J 2014; Weir and Goudet 2017; Taylor, Jacob, *et al.* 2019). Theoretically, we show via the nIBD-to-IBS model that the implicit embedding of population-averaged locuswise relatedness in standard observation models is pervasive (Weir and Goudet 2017) and can lead to the systematic underestimation of pairwise relatedness, especially when background relatedness is high. Larremore (2019) characterize conceptually concordant systematic underestimation of pairwise overlap between subsampled *P. falciparum var* gene repertoires. Guo *et al.* (2024) have shown that positive selection can also bias malaria parasite relatedness estimates, but selection-bias is minor when background relatedness is high. Beyond malaria, our theoretical results bear strong conceptual similarity to the work of Weir and Goudet (2017). Both theoretical and numerical analyses support the reinterpretation of pairwise relatedness under the independence model as a relative measure: non-zero estimates are calibrated intrinsically for relatedness averaged over the set of sampled parasites; zero estimates flag parasite pairs with below-average relatedness, but are otherwise uninformative (Hall *et al.* 2012; Weir and Goudet 2017). Numerically, we show that exploiting linkage structure using a HMM can mitigate bias in absolute relatedness estimates when genotypic data are sufficiently dense, and propose a dense data diagnostic to assess the severity of systematic bias. Using WGS *P. falciparum* data, we illustrate the use of the dense data diagnostic and characterize consequences of marker sparsity. Sparse data HMM estimates are difficult to interpret because systematic underestimation is only partially mitigated; nevertheless, threshold-based classification of HMM sparse data estimates is relatively robust.

The relevance of our findings is context-specific: the demand for absolute vs relative estimates varies on a case-by-case basis; the severity of systematic bias in absolute estimates depends on the average relatedness of sampled parasites, and thus on the sampling scheme and the parasite population; similarly, mitigation of bias under the HMM requires dense data and depends on linkage structure, which is population specific. In many malaria studies, data on most isolates are sparse, because sparse data are often of greater practical utility (Apinjoh *et al.* 2019; Noviyanti *et al.* 2020). Some studies complement sparse data analyses using WGS data on a subset of isolates. Others complement sparse data analyses using published WGS data from MalariaGEN, an invaluable community resource (Ahouidi *et al.* 2021). When all isolates have WGS data, practitioners can generate estimates at will, knowing bias under the HMM fit to WGS data is limited. For sparse data in isolation, HMM estimates can be categorized using high relatedness thresholds, or estimates can be generated under (n)IBD independence and interpreted relatively. When most data are sparse but there are some complementary (or preliminary) WGS data that are representative of the population of interest, the following three plots can help to decide between generating sparse-data estimates under (n)IBD independence or the HMM. Plot WGS (n)IBD independence estimates against WGS HMM estimates (dense data diagnostic) to assess whether caution around sparse-data HMM estimates is warranted. Plot downsampled (n)IBD independence estimates against WGS HMM estimates to assess the relative importance of bias vs uncertainty in sparse-data HMM estimates. Plot downsampled HMM estimates against WGS HMM estimates to identify thresholds above which highly-related parasite pairs can be identified sensitively.

### Limitations

#### IBS descriptives

The theoretical reinterpretation of pairwise relatedness under independence as a relative measure is predicated on IBS descriptives, as in Weir and Goudet (2017). In practice, pairwise relatedness is estimated using allelic states rather than IBS descriptives (Henden *et al.* 2018; Schaffner *et al.* 2018; Taylor, Jacob, *et al.* 2019; Zhu *et al.* 2019; Taylor *et al.* 2020; Taylor 2022a). Misspecification of the IBD-to-allele model—arising from the non-independence of IBD and allelic states—is sensitive to the unobservable relatedness structure at each locus. It is not necessarily rectified by removing replicates of clonal parasites, and may be particularly pronounced in the presence of selection (Guo *et al.* 2024). As such, there may be systematic differences between pairwise relatedness estimates based on IBS descriptives vs allelic states. A rigorous examination of the latter would potentially require joint estimation of relatedness across a set of sampled parasites, which is beyond the scope of the current manuscript.

#### Demographic processes

Demographic processes have a large effect on real data but are neither explored theoretically nor numerically, to avoid overburdening the manuscript. The effects of selection and immigration on the underlying relatedness structure of a set of sampled parasites are not considered. Our ancestral simulation model is very simple and does not recapitulate epidemiological reality. Transmission dynamics—which govern the propensity for selfing vs inbreeding vs outbreeding—are not explicitly modeled. We do not account for fluctuations in parasite population sizes, for instance, due to bottlenecks or control interventions, which may be particularly relevant in settings with high background relatedness.

#### Population structure

We did not explore population structure theoretically or numerically. In the presence of population structure, non-stratified sample allele frequencies can introduce further biases in relatedness estimates (Anderson and Weir 2007; Manichaikul *et al.* 2010; Rohlfs *et al.* 2012; Morrison 2013). Given variable levels of relatedness within and between populations, we posit the emergence of multiple elbows in the dense data diagnostic (see Appendix A.4 for an empirical example); however, we have not explored population structure thoroughly enough to know if a non-inbred but structured population could yield an elbow-like characteristic. Zero-inflation may not be present for cross-population comparisons with lower relatedness than the cross-population average. Our simulation model could be used to simulate different populations that unite and mix in order to interrogate the consequences of population structure. The simulation model of Guo *et al.* (2024), which accounts for population structure, could be used as an alternative or complementary approach. The framework of Weir and Goudet (2017), which jointly characterizes relatedness and population structure, could also be drawn on. Ancestry-informed estimation of sample allele frequencies can aid in mitigating biases due to population structure (Thornton *et al.* 2012; Moltke and Albrechtsen 2014). The cross-population model of hmmIBD implements a (n)IBD-to-allele model predicated on sample allele frequencies stratified by user-defined populations (Schaffner *et al.* 2018). It generates interpretable relatedness estimates in the presence of population structure, and could be analyzed to yield theoretical insight.

#### Multi-allelic markers

We derived results for multi-allelic markers in the absence of genotyping error, under the assumption that there are no systematic biological differences between multi-allelic and biallelic markers. That is to say, both are treated as nominal point polymorphisms, whose alleles can be modeled as categorical random variables (Taylor, Jacob, *et al.* 2019). We do not report our results on multi-allelic markers, because they are not meaningfully different to biallelic markers under these simplistic assumptions. In reality, the ancestral processes governing biallelic markers and multi-allelic markers likely differ. Multi-allelic markers additionally possess an ordinal genotyping error structure that is overlooked in current methods commonly used to estimate relatedness between malaria parasites (Henden *et al.* 2018; Schaffner *et al.* 2018). The practical ramifications could be explored using empirical data: qualitatively compare graphs of relatedness (as in Henden *et al.* 2018), where relatedness is estimated using multi-allelic markers (e.g. microhaplotypes) vs biallelic markers (e.g. SNPs) of equal informativeness (i.e. markers sets whose composite score of average effective cardinality multiplied by marker count is the same; see Taylor, Jacob, *et al.* 2019).

#### Data sparsity under simulation

We explore data sparsity using real data only. Our simulation model does not lend itself to the exploration of the effect of data sparsity: we intentionally elevate the recombination rate to compensate for computational constraints (small population size, few generations, single chromosome) and simplifying assumptions (no immigration, no mutation). Elevating the recombination rate generates simulated data whose estimates of relatedness resemble real data; it also leads to more IBD segments among simulated data which offsets the effect of data sparsity.

### Future work

#### Generating a community resource

The dense data diagnostic requires WGS data, which are often financially prohibitive (Apinjoh *et al.* 2019; Noviyanti *et al.* 2020). As a community resource, a catalogue of dense data diagnostic plots could be generated for published WGS datasets (Ahouidi *et al.* 2021). Doing so would first require partitioning samples into sets for which population structure is not a complicating factor.

#### Recurrent infection classification

Systematically elevated population relatedness likely impairs the resolution of recurrent infections during therapeutic efficacy studies. How best to deal with the problem is not yet understood. One existing approach involves embedding a relatedness inflation factor into a classification model (Taylor, Watson, *et al.* 2019). Our results suggest inflation factors in classification models that use sample allele frequencies are obsolete because sample allele frequencies already partially encode population-averaged locuswise relatedness.

#### Analyses using confidence intervals

A key consideration for sparse genotypic data is the significance of systematic bias in the presence of uncertainty attributable to marker sparsity. While we suggest downsampling loci to evaluate this trade-off heuristically, computing confidence intervals, as recommended in Taylor, Jacob, *et al.* (2019), may yield a more statistically principled approach.

#### Algorithmic correction of misspecification

Unbiased relatedness estimation using sparse data may be possible with joint inference of relatedness and the probability of allele sharing for nIBD parasites (Taylor, Jacob, *et al.* 2019). For instance, Hall *et al.* (2012) and Wang J (2022) adopt an iterative construction to jointly estimate IBD coefficients and allele frequencies, whereby these quantities are alternately readjusted across successive iterations; these methods could be adapted to the present context.

### Conclusion

Based on our results practitioners have two options: resolve to use relative relatedness estimated under independence or try to estimate absolute relatedness under a HMM. Because relative estimates are intrinsically adjusted, caution is required when differences across transmission settings are sought after. If relatedness estimates are used as relationship-indicators, relative values are arguably preferable across transmission settings. Caution should be exercised when estimating absolute relatedness using sparse data under a HMM because the extent to which underestimation is mitigated is unknowable, but classification of absolute values using high relatedness thresholds is relatively robust. We are reluctant to prescribe decision thresholds given most use cases are likely to deviate from any contrived examples. Instead, we provide tools to help practitioners evaluate their individual situations on a case-by-case basis.

## Supplementary Material

jkaf018_Supplementary_Data

## Figures and Tables

**Fig. A1.**
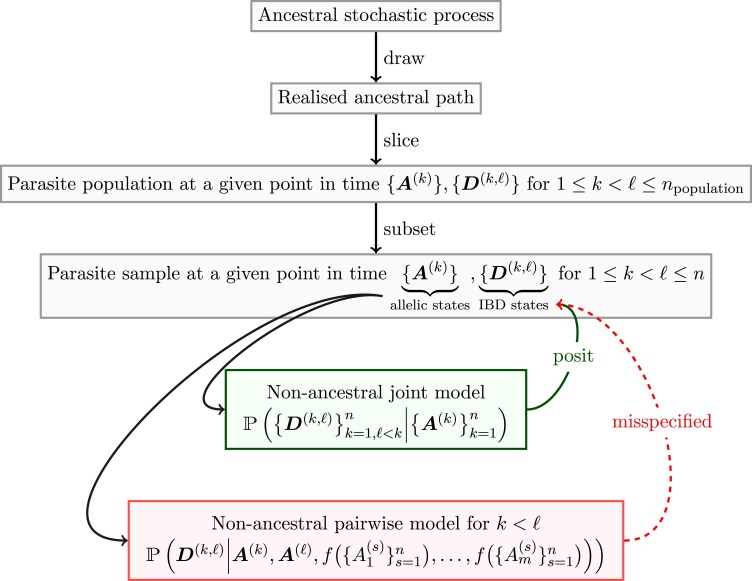
Conceptual overview, where $f(\{A_{i}^{\left(s\right)}{\}}_{s=1}^{n})$ denotes the vector of sample allele frequencies for alleles {1,…,y} for each locus i=1,…,m.

**Fig. A2.**
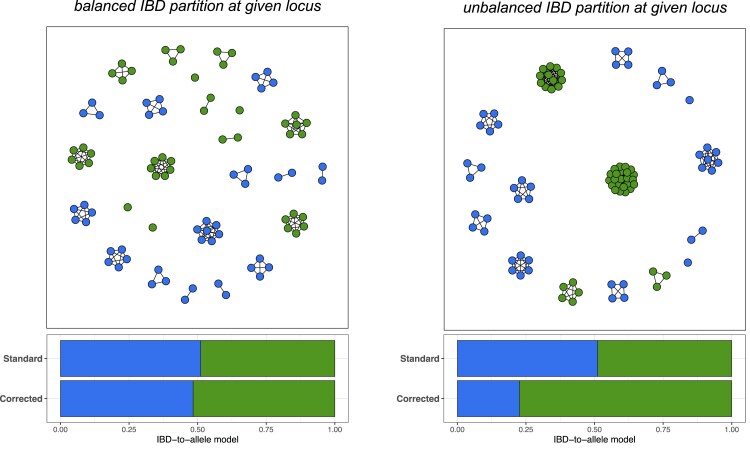
Schematic of misspecification under the standard IBD-to-allele model, relative to the proposed correction (A9), based on an underlying IBD network for a single locus. Each node represents an individual, colored by allelic state. Individuals that are IBD at that locus are connected by an edge. Since IBD is a transitive property (Taylor, Watson, *et al.* 2019), all components are fully connected. In the absence of genotyping error, edges can only be placed between nodes of the same color (that is, IBD necessarily implies IBS).

**Fig. A3.**
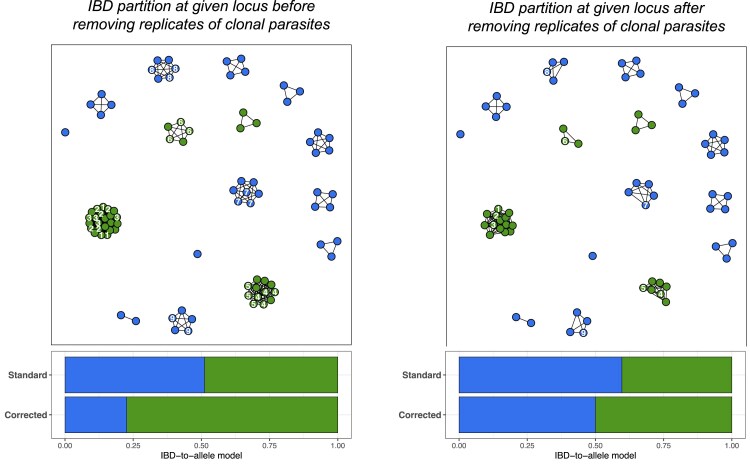
Unbalanced IBD partitions at a given locus before and after removing replicates of clonal parasites. Groups of clonal parasites are numbered 1–9.

**Fig. A4.**
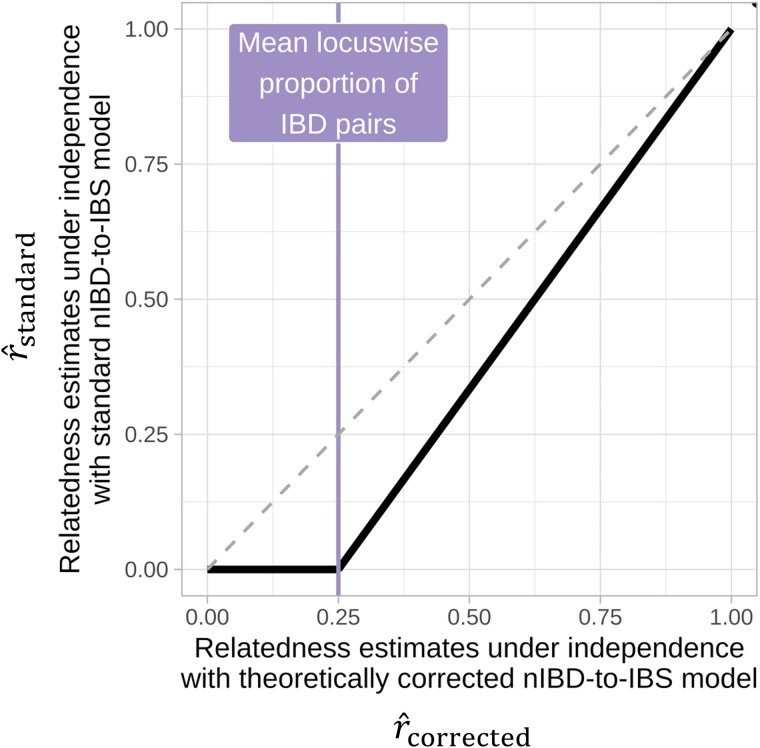
Schematic of MLEs of pairwise relatedness under the model of (n)IBD independence coupled with the standard (misspecified) model ${\hat{r}}_{\mathrm{standard}}$ vs corrected nIBD-to-IBS model ${\hat{r}}_{\mathrm{corrected}}$.

**Fig. A5.**
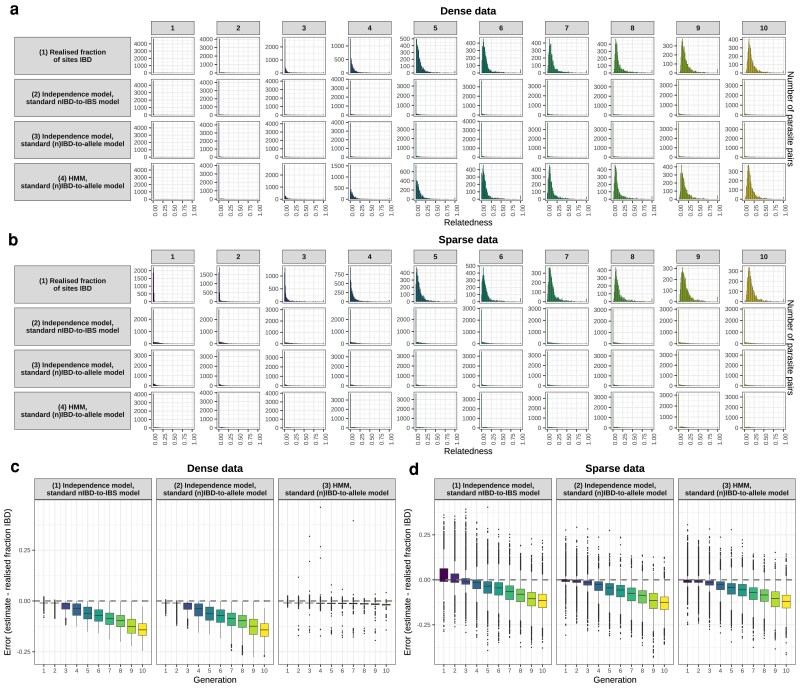
Pairwise relatedness estimates predicated on standard sample allele frequency-based observation models and either allelic states or IBS descriptives; either the independence model of relatedness or the HMM; and either dense or sparse data. Columns in subplots a) and b) correspond to the number of generations of inbreeding (1–10). Error, where applicable, is measured relative to the realized fraction of polymorphic sites IBD for each pairwise comparison. Sparse data have been obtained by selecting 200 polymorphic markers uniformly at random without replacement from the dense simulated dataset. Results are based a single realization of the simulation model, with parameter values as per Table B1.

**Fig. A6.**
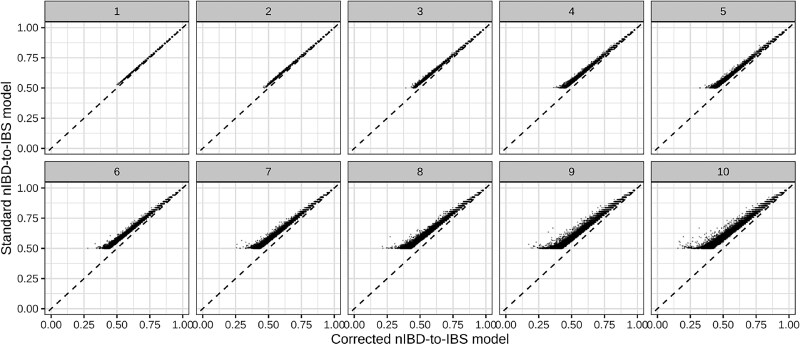
The locuswise proportion of IBS pairs $s_{i}$ (i.e. the standard nIBD-to-IBS model) vs the sample proportion of nIBD pairs that are IBS $c_{i}$ (i.e. the corrected nIBD-to-IBS model) across 10 successive generations of inbreeding (plots 1–10) for a single realization of the simulation model, with parameter values as per Table B1. For biallelic markers, it is necessarily the case that $s_{i}\geq 0.5$; however, $c_{i}$ may lie anywhere in the range [0,1].

**Fig. A7.**
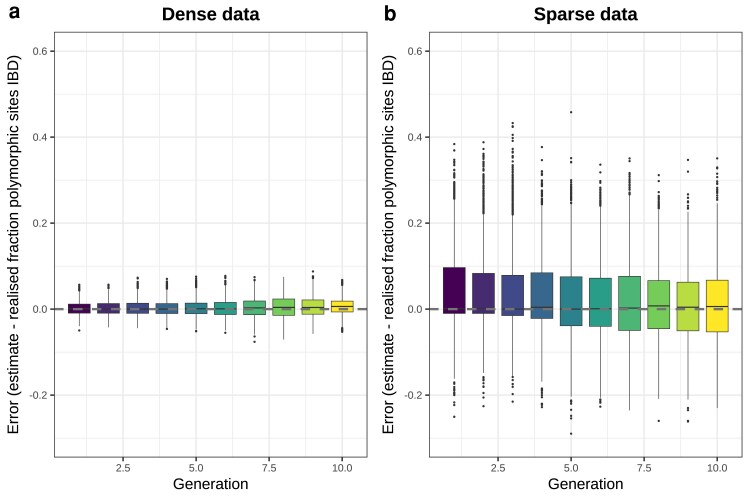
Relatedness estimates predicated on the independence model and the corrected locuswise nIBD-to-IBS model $c_{i}$. Sparse data have been obtained by selecting 200 polymorphic markers uniformly at random without replacement from the dense simulated dataset. Results are based a single realization of the simulation model, with parameter values as per Table B1.

**Fig. A8.**
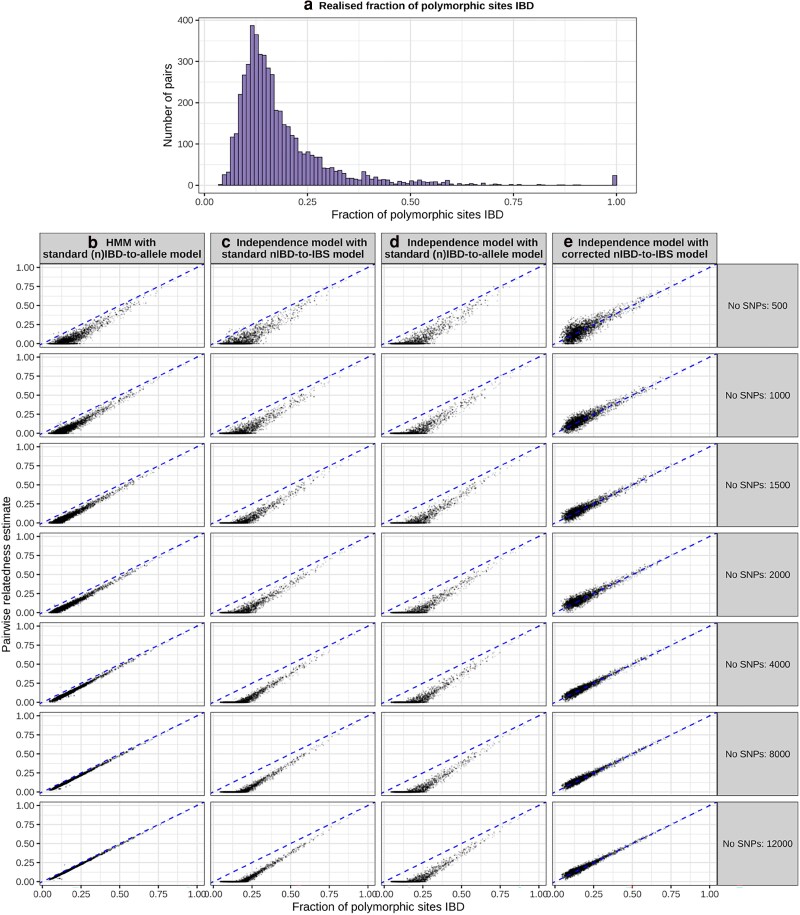
Summary of pairwise relatedness for a single realization of the simulation model after 10 generations of inbreeding (with parameter values as per Table B1), yielding the distribution of realized relatedness (i.e. the fraction of simulated polymorphic sites that are IBD for each parasite pair) shown in a). For downsampled marker subsets (selected uniformly at random without replacement over the set of polymorphic markers), realized relatedness is calculated over the marker subset and treated as the ground truth. We compare realized relatedness against pairwise relatedness estimates generated under b) the HMM with the standard (n)IBD-to-allele model; c) (n)IBD independence with the standard (n)IBD-to-allele model; d) (n)IBD independence with the standard nIBD-to-IBS model; e) (n)IBD independence with the theoretically corrected nIBD-to-IBS model.

**Fig. A9.**
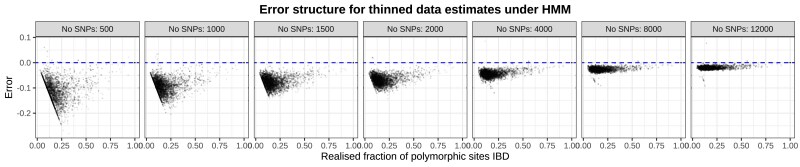
Error structure for relatedness estimates of downsampled marker subsets (selected uniformly at random without replacement over the set of polymorphic markers) at generation 10 under the HMM of relatedness with the standard (n)IBD-to-allele model, compared with the realized fraction of polymorphic sites IBD for each parasite pair. Results are based on a single realization of the simulation model, with parameter values as per Table B1.

**Fig. A10.**
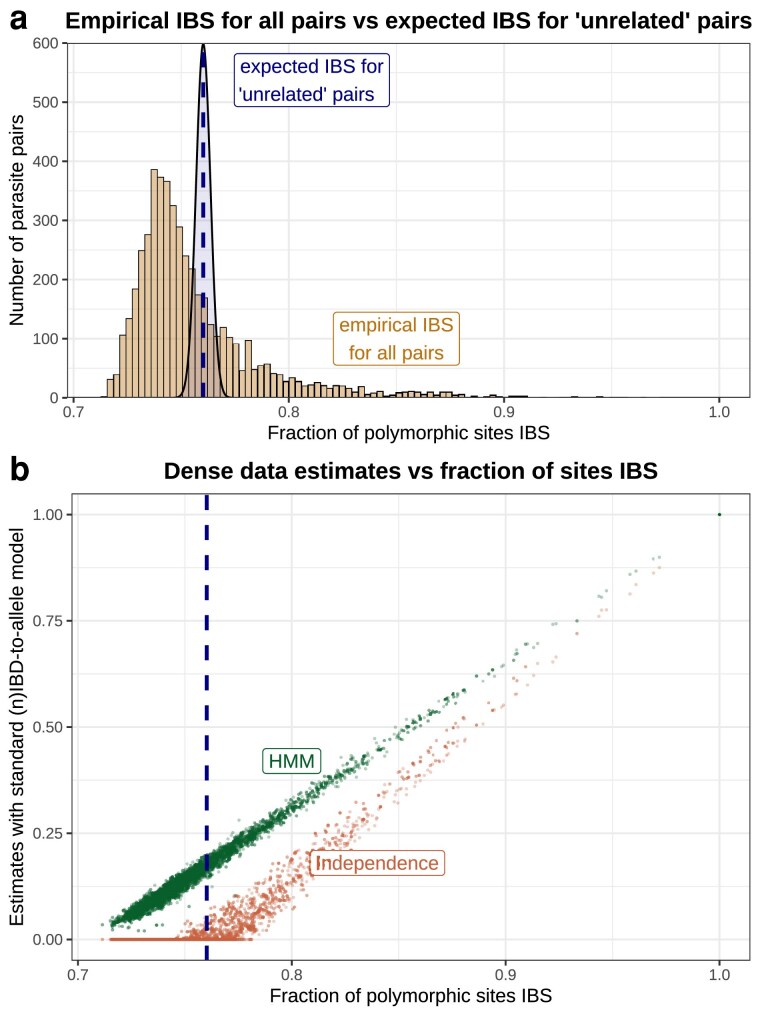
For dense data at generation 10: a) The empirical pairwise distribution of IBS sharing for all pairs (ochre), defined to be the fraction of polymorphic sites that are IBS, compared with the expected IBS distribution $Y_{0}$ for ostensibly unrelated pairs (blue). b) Relatedness estimates generated under the HMM (green) or independence model (orange) using the standard (n)IBD-to-allele model vs the fraction of polymorphic sites IBS. Results are based on a single realization of the simulation model, with parameter values as per Table B1. The probability mass function for the Poisson binomial distribution has been computed using the R package poisbinom (Olivella and Shiraito 2017).

**Fig. A11.**
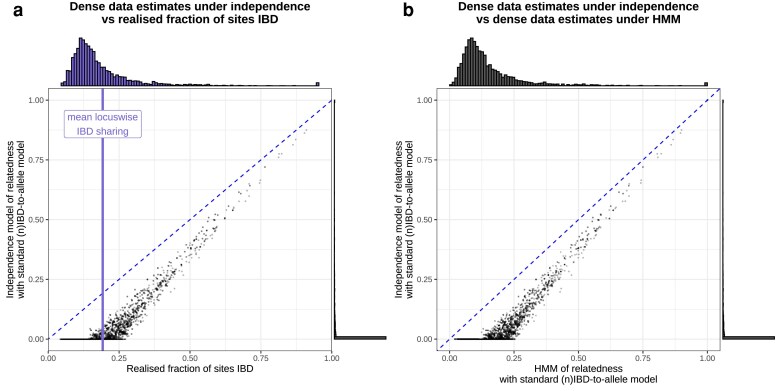
Relatedness estimates for dense genotypic data at generation 10 under the independence model vs a) the realized fraction of polymorphic sites IBD or b) corresponding estimates under the HMM of relatedness. Here, we use the standard (n)IBD-to-allele model. Results are based on a single realization of the simulation model, with parameter values as per Table B1.

**Fig. A13.**
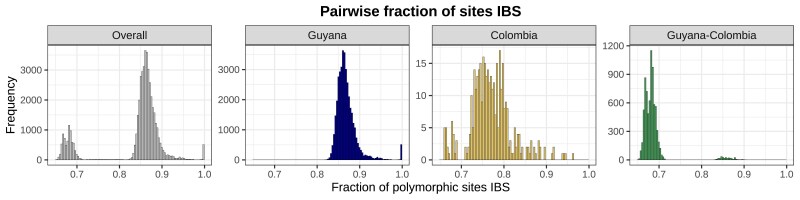
Multimodality in distributions of pairwise fractions of IBS markers. In the light of missing data, IBS sharing for each pair is defined to be the fraction of polymorphic sites that are not missing data and are IBS. We stratify comparisons within and between countries.

**Fig. A14.**
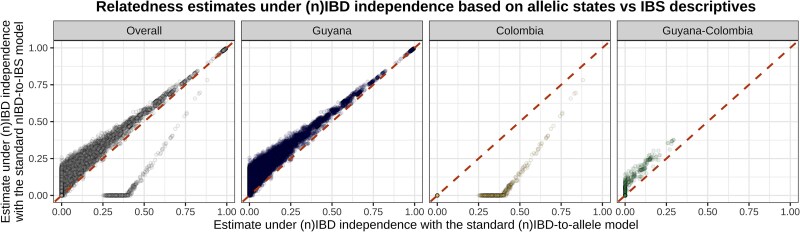
Comparison of relatedness estimates generated under (n)IBD independence using the standard nIBD-to-IBS model vs the standard (n)IBD-to-allele model as a manifestation of population structure. We stratify comparisons within and between countries.

**Fig. B1.**
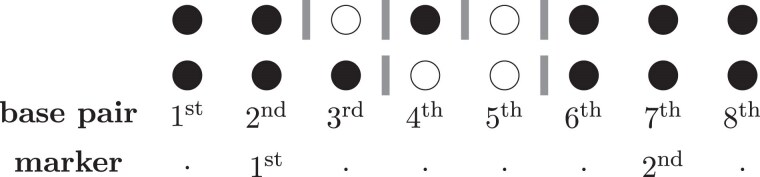
Two examples of pairwise (n)IBD states across a stretch of 8 bp. Recombination breakpoints (vertical bars) are shown between IBD (black) and nIBD (white) loci. Consecutive IBD markers may be separated by an even number of recombination breakpoints, that may not be detectable due to the discrete sampling of the genome.

**Fig. B2.**
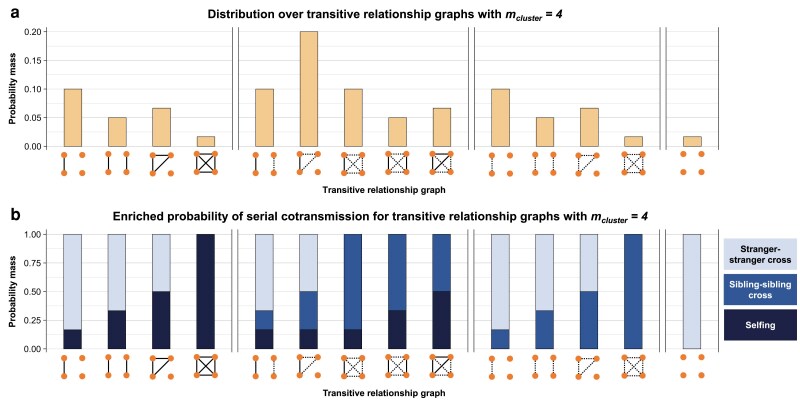
a) Probability masses for transitive relationship graphs with sibling/clone/stranger edges (Taylor, Watson, *et al.* 2019) in the case $m_{\mathrm{subgraph}}=4$ (after removing node labels), under a uniform distribution over all allowable graphs (see Appendix B.3.1). Clonal and sibling edges are indicated with solid and dashed lines, respectively. b) Probability of stranger–stranger crosses vs sibling–sibling crosses vs selfing when two parents are sampled uniformly at random *without* replacement from each allowable relationship graph with $m_{\mathrm{subgraph}}=4$.

## Data Availability

Sequencing data for *P. falciparum* isolates from both Guyana (Vanhove *et al.* 2024) and Colombia (Carrasquilla *et al.* 2022) are available in the NCBI Sequencing Read Archive; accession numbers are provided in supplementary Table S1. A minimum analysis dataset for the present project, comprising a genotype matrix, is available on the accompanying GitHub repository (Mehra *et al.* 2024): https://github.com/somyamehra/PlasmodiumRelatednessBias  https://doi.org/10.5281/zenodo.14176553. Supplemental material available at G3 online.

## Appendix

### Appendix A: Detailed methods and results

**Table A1.** Overview of results, with theoretic, numerical, and/or empirical support.

	Theory	Numerical	Empirical
**Sample allele frequencies partially encode relatedness structure** *Misspecification of standard (n)IBD-to-observation models*	✓		
**Relatedness is systematically underestimated under the independence model** *Re-interpretation as a relative measure, intrinsically adjusted for relatedness averaged over the parasite sample; zero-inflation*	✓	✓	
**Exploiting linkage structure using the HMM of relatedness can mitigate underestimation for dense datasets** *Reduced sensitivity to misspecified (n)IBD-to-observation models*		✓	
**A dense data diagnostic: gauging the severity of underestimation** *Comparison of dense data estimates under the independence model vs HMM can elucidate average population-level relatedness*		✓	✓
**Analysing sparse data under the HMM yields an intermediary regime** *Partial but incomplete mitigation of underestimation; may be sufficient to identify highly related parasite pairs*			✓
**Additional manifestations of population structure** *Multimodal empirical IBS distributions and systematic differences in relatedness estimates with IBS descriptives vs allelic states indicate population structure*			✓

#### A.1. Methods

Our approach for characterizing systematic biases in malaria parasite relatedness estimation is 3-fold. We begin by deriving results under a theoretical framework. We then construct a simulation model under which we verify our theoretical results and design practical diagnostics. We conclude with a case study of *P. falciparum* data. Throughout, individual is used to refer to a parasite genotype drawn from an infected host, whereas sample is used to refer to a collection of k=1,…,n parasite genotypes drawn from many infected hosts. See Table A2 for a complete list of notation used throughout this appendix. For brevity and clarity of exposition, this notation differs from the main text: specifically, the sample proportion of pairs that are IBD (IBS) at locus *i* is denoted $d_{i}$ ($s_{i}$) here, compared with ${\bar{\text{IBD}}}_{i}$ (${\bar{\text{IBS}}}_{i}$) in the main text.

**Table A2.** Summary of notation and key quantities for Appendix A only.

Quantity	Interpretation	Equation
$A_{i}^{\left(k\right)}$	Allelic state for individual *k* at locus *i* (observable)	–
$S_{i}^{\left(k,\ell \right)}$	IBS state for individuals k,ℓ at locus *i* (observable)	(A2)
$D_{i}^{\left(k,\ell \right)}$	IBD state for individuals k,ℓ at locus *i* (unobservable)	–
$f_{i}(q)$	Sample frequency of allele *q* at locus *i* (observable)	(A1)
$d_{i}$	Sample proportion of pairs IBD at locus *i* (unobservable)	(A8)
$s_{i}$	Sample proportion of pairs IBS at locus *i* (observable)	(A12)
$c_{i}$	Sample proportion of pairs IBC at locus *i* (unobservable)	(A13)
$r^{\left(k,\ell \right)}$	Pairwise relatedness parameter for parasites *k*, ℓ	(A19)
${\hat{r}}^{\left(k,\ell \right)}$	MLE of pairwise relatedness parameter for parasites *k*, ℓ	–

The default for comparative variables includes self–self comparisons (that is, entails sampling with replacement).

##### A.1.1 Theoretical framework

While allelic concordance or identity-by-state (IBS) is observable, it can be attributed to one of two latent states: identity-by-descent (IBD), reflecting a common ancestral origin; identity-by-chance (IBC), otherwise. Hereafter, we use nIBD as shorthand for “not IBD”. Note that IBC≠nIBD; rather, IBC=IBS∩nIBD. We use (n)IBD as shorthand for both IBD and nIBD, likewise for (n)IBS.

Before proceeding, let us introduce some notation. Given a sample of *n* not necessarily distinct parasite genotypes, we index each genotype k=1,…,n. We allow each locus i=1,…,m to harbor an allele in the set {1,…,y}, where *y* denotes the maximal cardinality. We denote by $A_{i}^{\left(k\right)}$ the allele observed at locus *i* in genotype *k*. The sample frequency of allele *q* at locus *i*

(A1 )
$$f_{i}(q)=\frac{1}{n}\sum_{k=1}^{n}1\{A_{i}^{\left(k\right)}=q\},$$

where 1{⋅} denotes the indicator function. For a given parasite pair (k,ℓ), we further define

- $S_{i}^{\left(k,\ell \right)}=1$ if locus *i* is IBS for the pair (k,ℓ) and zero otherwise,
  (A2 ) $$S_{i}^{\left(k,\ell \right)}=1\{A_{i}^{\left(k\right)}=A_{i}^{\left(\ell \right)}\}=\sum_{q=1}^{y}1\{A_{i}^{\left(k\right)}=q\}1\{A_{i}^{\left(\ell \right)}=q\}.$$
- $D_{i}^{\left(k,\ell \right)}=1$ if locus *i* is IBD for the pair (k,ℓ) and zero otherwise.

Throughout, all pairwise comparisons include self–self comparisons.

Relatedness structure within a parasite population is governed by some unknown ancestral stochastic process, replete with demographic complexity. The characterization and analysis of this ancestral process is beyond our scope. Instead, we seek to analyze a sample of parasite genotypes at a given point in time using a non-ancestral model. In particular, given $\{A^{\left(k\right)}{\}}_{k=1}^{n}$ for a sample of *n* individuals, we seek to estimate $\{D^{\left(k,\ell \right)}{\}}_{k=1,\ell <k}^{n}$ (Fig. A1).

##### A.1.1.1 Joint model of malaria parasite relatedness

In an ideal setting, we would perform joint inference over the sample of *n* individuals (Wang 2004; Taylor, Jacob, *et al.* 2019). The construction of a joint model, however, is nontrivial. The principal complication lies in the nonindependence of IBD states $D^{\left(k,\ell \right)}$ between pairs of individuals. Since IBD is a transitive property, rather than considering parasite pairs in isolation, we would need to reconstruct the complete relatedness structure of a population: a system of correlated, transitive graphs—each representing IBD sharing at a given locus (Taylor, Watson, *et al.* 2019)—with the number of allowable configurations per graph growing as Bell numbers as a function of the parasite population size. This poses a significant combinatorial problem, quickly rendering brute force approaches computationally intractable (Taylor, Watson, *et al.* 2019).

##### A.1.1.2 Non-ancestral pairwise Markov model of (n)IBD states

In practice, relatedness is estimated pairwise using a non-ancestral model, comprising an alternating Poisson process (APP) (Stam 1980). For a given parasite pair, we conceptualize the genome as a mosaic of alternating (n)IBD segments, with the intuition that IBD tracts are fragmented by randomly distributed recombination breakpoints over successive generations (Stam 1980).

While the genome is treated to be continuous under the APP model, we sample a finite number of discrete loci i=1,…,m. To describe the sequence of IBD states

$$D=(D_{1},\ldots ,D_{m})\in \{0,1{\}}^{m}$$

across loci i=1,…,m (where parasite identifiers k,ℓ are dropped for notational convenience), Leutenegger *et al.* (2003) construct a discrete-time Markov process. Linkage between successive markers is parameterized by:

- the genomic distance $\delta_{i}$ in units of base pairs (bp) between loci *i* and (i+1);
- a constant recombination rate *ρ* in units of Morgans per base pair (M/bp), typically ascertained from genetic cross experiments (Jiang *et al*. 2011; Miles *et al.* 2016); and
- a (n)IBD latent state switching rate *κ* that is unobservable and must be inferred.

We assume that locus 1 is IBD with probability *r*, that is,

$$P(D_{1}=1)=r,\;P(D_{1}=0)=1-r.$$

Under the Markov property, $D_{i+1}$ is dependent only on $D_{i}$. The complete sequence of IBD states D is governed by the transition matrix

(A3 )
$$\begin{array}{rl} & \left(\begin{array}{cc} P(D_{i+1}(t)=0\;|\;D_{i}(t)=0) & P(D_{i+1}(t)=1\;|\;D_{i}(t)=0)\\ P(D_{i+1}(t)=0\;|\;D_{i}(t)=1) & P(D_{i+1}(t)=1\;|\;D_{i}(t)=1) \end{array}\right)\\ & \;\;:\;=\left(\begin{array}{cc} 1-r(1-e^{-\kappa \rho \delta_{i}}) & r(1-e^{-\kappa \rho \delta_{i}})\\ \left(1-r)(1-e^{-\kappa \rho \delta_{i}}\right) & 1-(1-r)(1-e^{-\kappa \rho \delta_{i}}) \end{array}\right), \end{array}$$

as per Taylor, Jacob, *et al.* (2019), where *k* was used instead of *κ* for the switch rate parameter. The degree of dependence between successive loci is governed by the product $\kappa \rho \delta_{i}$: to weaken the dependence between successive loci, we can either increase the switching rate *κ*, or the genomic distance $\delta_{i}$ between successive loci.

##### A.1.1.3 Non-ancestral pairwise independence model of (n)IBD states

Under the above-mentioned non-ancestral Markov model in the limit κ→∞ (Taylor, Jacob, *et al.* 2019) or $\delta_{i}\to \infty$ (Taylor *et al.* 2017), we recover the independence model

(A4 )
$$P(D)=\prod_{i=1}^{m}r^{D_{i}}(1-r{)}^{1-D_{i}},$$

where locuswise (n)IBD states are described by independent and identically distributed Bernoulli random variables with success probability *r*.

##### A.1.1.4 Observation models

Since (n)IBD states are unobservable, estimation of the pairwise relatedness parameter *r* necessitates coupling the model of hidden (n)IBD states to a model of observations conditional on (n)IBD states. Herein, observations are either alleles or (n)IBS descriptives (Box A1). We do not model genotyping errors. Elsewhere, where genotyping errors are taken into account (e.g. Taylor, Jacob, *et al.* 2019), the observation model integrates over latent nonerroneous alleles and is thus made of modules: an (n)IBD-to-latent-allele observation model, and a model capturing genotyping error, e.g.

$$P\left(A_{\mathrm{obs}}^{\left(k\right)},A_{\mathrm{obs}}^{\left(\ell \right)}\;|\;D^{\left(k,\ell \right)}\right)=\sum_{A^{\left(k\right)},A^{\left(\ell \right)}}\underset{\begin{array}{c} (n)\mathrm{IBD}-\mathrm{to}-\mathrm{latent}-\mathrm{allele}\\ \;\mathrm{model} \end{array}}{\underbrace{P\left(A^{\left(k\right)},A^{\left(\ell \right)}\;|\;D^{\left(k,\ell \right)}\right)}}\;\;\;\;\;\;\times \underset{\mathrm{error}\;\mathrm{model}}{\underbrace{P\left(A_{\mathrm{obs}}^{\left(k\right)},A_{\mathrm{obs}}^{\left(\ell \right)}\;|\;A^{\left(k\right)},A^{\left(\ell \right)}\right)}}.$$

Further details of the observations models are provided in the results section (Appendix A.2). They include standard practice observation models (models into which sample allele frequencies are plugged, where sample allele frequencies may be computed before or after removing replicates of seemingly clonal parasites); and a corrected model of independent (n)IBS states, which is not practically available, but useful for validating theory when applied to simulated data.

Box A1: Observations are either alleles or (n)IBS descriptives Consider the following data on a pair of individual genotypes
*genotype 1*: ATCG
*genotype 2*: ACCG The observations on the pair of genotypes 1 and 2 are

**Table:**

(A,A)	(T,C)	(C,C)	(G,G)	using alleles
IBS	nIBS	IBS	IBS	using (n)IBS descriptives

##### A.1.2 Simulated data

The model under which simulated data are generated is described in detail in Appendix B. Assumed parameter values, and their respective interpretations, are detailed in Table B1. The model’s purpose is not to recapitulate epidemiological reality, but to generate data that can be used to verify theoretical results and facilitate the design of practical diagnostics. To ensure verification is independent, data are simulated under a model built on ancestral principles; they are not simulated under the non-ancestral models used to estimate relatedness.

Under the simulation model, there is a dichotomy between ancient low-level background relatedness stemming from generation zero, and very recent relatedness arising from recent breeding under a small fixed population size. To balance the distribution of IBD segment lengths in spite of this dichotomy, we use a recombination rate and genome size that substantially exceeds that of *P. falciparum* (Miles *et al.* 2016).

Estimates of pairwise relatedness generated under the non-ancestral relatedness models are compared with true pairwise relatedness values, which are known for simulated data. For a given pair of simulated individuals, we use an approximation of realized relatedness as our truth value, where realized relatedness is the fraction of loci that are IBD (Speed and Balding 2015). Under our simulation model where markers are equidistant, realized relatedness is approximated by the fraction of polymorphic markers that are IBD: IBD is ascertained by comparing ancestral founder mosaics for each pair of simulated individuals; polymorphic markers are those at which there is some variation within the sample, noting that the number of polymorphic markers for a given sample tends to decrease over generations due to loss of diversity. We compute realized relatedness using polymorphic markers because pairwise relatedness estimates are predicated on data from polymorphic markers only, and there may be increasing variability as the set of polymorphic markers is thinned over generations.

##### A.1.3 P. falciparum data

We illustrate the practical consequences of our theoretical and numerical findings through a case study. We focus on a set of highly quality isolates from passively sampled symptomatic patients in Guyana in 2016–2020 (Vanhove *et al.* 2024), as well as from Colombia in 1993–2017 (Carrasquilla *et al.* 2022), that are deemed to be monoclonal. WGS data for these isolates were processed previously in accordance with GATK best practices to yield a genomewide set of variants as described in Carrasquilla *et al.* (2022) and Vanhove *et al.* (2024); additional filtration criteria are detailed in the main text. This yields a WGS SNP dataset comprising n=306 isolates (n=278 from Guyana, n=28 from Colombia) and n=30,694 polymorphic biallelic SNPs. Using the dataset, we elucidate the consequences of population structure, which is omitted from our theoretical and numerical analyses. We also illustrate the implications of marker sparsity on pairwise relatedness estimates for n=278 isolates from Guyana (n=16,115 polymorphic biallelic SNPs), generating sparse marker panels by down-sampling SNPs sites uniformly at random without replacement.

#### A.2 Theoretical results

Standard (n)IBD-to-observation models are constructed using sample allele frequencies. When relatedness structure is significant, overlooking it in allele frequency estimation can introduce significant biases (Rousset 2002; Wang J 2002, 2011, 2014, 2022; Wang 2004; Yu *et al.* 2006; Bink *et al.* 2008; Kang *et al.* 2010; Weir and Goudet 2017), with practical consequences including the systematic underestimation of relatedness (Bink *et al.* 2008) (Case A, Box A2). Removing replicates of seemingly clonal parasites from the sample before computing allele frequencies re-weights allele frequencies in a case-specific manner. It does not account for relatedness between the remaining sample members, however. As such, systematic underestimation of relatedness is liable to persist (Case A, Box A2). An alternative naive approach, evoking the assumption of equifrequent alleles, may yield systematic overestimates of pairwise relatedness (Bink *et al.* 2008) (Case B, Box A2).

Box A2: Some consequences of misspecified observation models
***Likelihood of pairwise IBS sharing for a given locus assuming no error***

$$\begin{array}{rl} P(\text{IBS}) & =P(\text{IBS}\;|\;\text{IBD})P(\text{IBD})+P(\text{IBS}\;|\;\text{nIBD})P(\text{nIBD})\\ & =P(\text{IBD})+P(\text{IBS}\;|\;\text{nIBD})\left(1-P(\text{IBD})\right) \end{array}$$

since P(IBS|IBD)=1 (in the absence of genotyping error), and where P(IBD) is a genomewide measure of relatedness.
***Implications for relatedness estimates***

- P(IBS|nIBD) overestimated ⟹ ⇒ P(IBD) underestimated
- P(IBS|nIBD) underestimated ⟹ ⇒ P(IBD) overestimated

***Observation models***

(A) $P(\text{IBS}\;|\;\text{nIBD})\approx \bar{\text{IBS}}$, the observed proportion of IBS pairs in the ${\mathrm{sample}}^{a}$
  **Table:**
  *Calculation:*	sums of squares of sample allele frequencies
  *Possible issue:*	IBS due to IBD is not attributed to IBD
  *Implication:*	P(IBS|nIBD) may be overestimated
(B) P(IBS|nIBD)≈1/n where *n* is locus cardinality
  **Table:**
  *Calculation:*	reciprocal of observed allele count
  *Possible issue:*	alleles are not equifrequent
  *Implication:*	P(IBS|nIBD) may be underestimated (Munford 1977)

^a^Replicates of clonal parasites may have been removed from the sample or not

Starting with the standard (n)IBD-to-allele model, and its corrected IBD-to-allele counterpart (Appendix A.2.1), we show that standard observation models can be misspecified given both IBD (Appendix A.2.1.1) and nIBD (Appendices A.2.1.2, A.2.2.1, and A.2.2.2), and reason why removing replicates of seemingly clonal parasites before computing allele frequencies rectifies neither case. More specifically, our theoretical results show that, given IBD, standard observation models are potentially misspecified due to assumed independence between IBD and allelic states, which does not always hold (Appendix A.2.1.1); given nIBD they are misspecified due to partial encoding of the locuswise proportion of IBD pairs within the sums of squares of sample allele frequencies (Weir and Goudet 2017) (Appendix A.2.1.2). Systematic underestimation of pairwise relatedness may be associated with this misspecification. Under the independence model of relatedness with IBS descriptives, we explore additional consequences of this misspecification (Appendix A.2.2). Firstly, echoing the work of Weir and Goudet (2017) (who also adopt IBS descriptives), we re-interpret the pairwise relatedness parameter *r* as a relative measure, capturing deviation from average relatedness (Appendix A.2.2.1). Secondly, we show pairwise relatedness estimates are stratified by average relatedness, with zero-valued estimates below and positive estimates above (Appendix A.2.2.2), likewise echoing Weir and Goudet (2017). While our theoretical results are restricted to the independence model, the implications of marker density and linkage are explored through analyses of simulated and empirical data (Appendices A.3 and A.4, respectively).

##### A.2.1 The standard (n)IBD-to-allele model and its corrected IBD-to-allele counterpart

The construction of the standard (n)IBD-to-allele model (Henden *et al.* 2018; Schaffner *et al.* 2018; Taylor, Jacob, *et al.* 2019; Taylor *et al.* 2020) is two-fold:

1. Given a pair of parasites (k,ℓ) is IBD at locus *i*, the probability that both individuals have allele *q* is the sample frequency of allele *q* at locus *i*:
  (A5 ) $$P_{\mathrm{standard}}(A_{i}^{\left(k\right)}=A_{i}^{\left(\ell \right)}=q\;|\;D_{i}^{\left(k,\ell \right)}=1)=f_{i}(q).$$
2. Given a pair of parasites (k,ℓ) is nIBD at locus *i*, the probability of observing alleles (q,u) is the product of the sample frequencies of alleles *q* and *u* at locus *i*:
  (A6 ) $$P_{\mathrm{standard}}(A_{i}^{\left(k\right)}=q,A_{i}^{\left(k\right)}=u\;|\;D_{i}^{\left(k,\ell \right)}=0)=f_{i}(q)\cdot f_{i}(u),$$
  where $f_{i}$ is a function of 1,…,n individuals (Equation (A1)).

In other words, it is assumed that allele frequencies, when restricted to IBD pairs, are equivalent to sample allele frequencies (Equation (A5)); and the likelihood of each pairwise allelic state, when restricted to non-IBD pairs, is equivalent to the sample incidence of that pairwise allelic state (Equation (A6)).

This description applies when allele frequencies are computed using samples from which replicates of seemingly clonal parasites have been removed or not. As such, all results that follow apply to either case. The only difference between the cases is the specific values of $f_{i}(q)$, $s_{i}$, $d_{i}$ etc., which will differ on a case-by-case basis not amenable to generalization.

To highlight the misspecification of standard observation models, it is instructive to introduce some unobservable quantities of interest:

- The sample proportion of pairs IBD at locus *i* harboring allele *q*
  (A7 ) $$d_{i}(q)=\frac{1}{n^{2}}\sum_{k=1}^{n}\sum_{\ell =1}^{n}1\{A_{i}^{\left(\ell \right)}=q\}\cdot D_{i}^{\left(k,\ell \right)}$$
  in which the indicator features only once since $D_{i}^{\left(k,\ell \right)}=1$ implies $A_{i}^{\left(k\right)}=A_{i}^{\left(l\right)}$.
- The sample proportion of pairs IBD at locus *i*, i.e. the locuswise average relatedness
  (A8 ) $$d_{i}=\sum_{q=1}^{y}d_{i}(q)=\frac{1}{n^{2}}\sum_{k=1}^{n}\sum_{\ell =1}^{n}D_{i}^{\left(k,\ell \right)}.$$

The sample proportion of individuals that are IBD at locus *i* and harbor allele *q* is $d_{i}(q)/d_{i}$. If it were observable, $d_{i}(q)/d_{i}$ would be used to approximate the true probability of an IBD pair harboring allele *q* under an IBD-to-allele model, since we would expect the sample proportion to converge to the true probability as the sample size *n* goes to infinity, at least under the conventional law of large numbers. As such, we claim that

(A9 )
$$P_{\mathrm{corrected}}(A_{i}^{\left(k\right)}=A_{i}^{\left(\ell \right)}=q\;|\;D_{i}^{\left(k,\ell \right)}=1)\approx \frac{d_{i}(q)}{d_{i}}.$$

##### A.2.1.1 The standard IBD-to-allele model assumes independence between IBD and allelic states

When IBD and allelic states are independent, $d_{i}(q)=f_{i}(q)\cdot d_{i}$, rendering

(A10 )
$$P_{\mathrm{corrected}}(A_{i}^{\left(k\right)}=A_{i}^{\left(\ell \right)}=q\;|\;D_{i}^{\left(k,\ell \right)}=1)\approx f_{i}(q).$$

Otherwise stated, the standard IBD-to-allele model (Equation (A5)) assumes independence between allelic and IBD states. If independence between allelic and IBD states does not hold, then the sample allele frequency $f_{i}(q)$ is not necessarily a good approximation of the true probability of an IBD pair harboring allele *q*, and the standard IBD-to-allele model is misspecified.

The presence of relatedness structure within the sample can violate the assumption of independence between allelic and IBD states. By relatedness structure, we refer to the locuswise partitioning of individuals into IBD clusters (Taylor, Watson, *et al.* 2019). If subcluster sizes are equal, then Equation (A5) will hold. Unbalanced subcluster sizes, however, can lead to substantial divergence between the sample proportion of IBD pairs harboring each allelic state and the likelihood under the standard IBD-to-allele model (Equation (A5)) (e.g. Fig. A2); this may occur if a particular allele is under strong positive selection. In the context of sibship reconstruction, which likewise involves partitioning samples by ancestry, the correction of sample allele frequencies is posited to be particularly pertinent in small populations with variable family/partition sizes (Thomas and Hill 2000; Smith *et al*. 2001); an analogous prescription applies here. Unlike family size, relatedness structure within a parasite population is unobservable and poses significant combinatorial challenges for inference: given dependence between parasite pairs, inference of relatedness, and allele frequencies should be performed jointly over parasite population. Without due consideration of underlying demographic processes and an associated mechanistic model of ancestry, it is difficult to gauge the expected relatedness structure within a population. Diagnosing the degree of misspecification under the model likelihood (Equation (A5)) is therefore nontrivial in practice.

Besides in the unrealistic scenario where the parasite population is split into unrelated clonal clusters, removing replicates of seemingly clonal parasites will not remove replicates within locuswise IBD partitions because individuals can be IBD at a given locus without being IBD at all loci (Fig. A3). Otherwise stated, removing replicates of seemingly clonal parasites from a sample does not remove relatedness between the remaining sample members.

##### A.2.1.2 The standard nIBD-to-IBS model encodes locuswise average relatedness

The observation model of allelic states for nIBD loci (Equation (A6)) is misspecified in the conflation of IBC and IBS: an unobservable and unaccounted proportion of IBS is driven by IBD. Similarly to Weir and Goudet (2017), we shift to IBS descriptives hereafter to:

1. Circumvent misspecification arising from the assumed independence of allelic and IBD states implicit in Equation (A5).
2. Highlight the confounding effects of relatedness structure in the construction of observation models for nIBD pairs (Equation (A6)).

Condensing Equation (A6) to consider only IBS descriptives yields the following nIBD-to-IBS model:

(A11 )
$$P_{\mathrm{standard}}(S_{i}^{\left(k,\ell \right)}=1\;|\;D_{i}^{\left(k,\ell \right)}=0)=\sum_{q=1}^{y}f_{i}{\left(q\right)}^{2}=\;:\;s_{i}.$$

We note that Equation (A11) is precisely Nei’s gene identity metric (Nei 1973; Taylor, Jacob, *et al.* 2019). We can alternatively interpret $s_{i}$ as an estimate of the probability that two identical alleles are selected from a pool of *n* alleles $A_{i}^{\left(k\right)}$, k=1,…,n  *with* replacement by rearranging Equation (A11) to obtain the expression

(A12 )
$$\begin{array}{rl} s_{i}\;: & =\sum_{q=1}^{y}{\left(\frac{1}{n}\sum_{k=1}^{n}1\{A_{i}^{\left(k\right)}=q\}\right)}^{2}\\ & =\frac{1}{n^{2}}\sum_{k=1}^{n}\sum_{\ell =1}^{n}\left(\sum_{q=1}^{y}1\{A_{i}^{\left(k\right)}=A_{i}^{\left(\ell \right)}=q\}\right)\\ & =\frac{1}{n^{2}}\sum_{k=1}^{n}\sum_{\ell =1}^{n}S_{i}^{\left(k,\ell \right)}, \end{array}\;$$

where we have substituted Equations (A1) and (A2) and interchanged the order of summation and products.

In the absence of genotyping error, such that locus *i* can only be IBD if it is IBS, that is, $S_{i}^{\left(k,\ell \right)}\geq D_{i}^{\left(k,\ell \right)}$, the sample proportion of nIBD individuals that are IBS at locus *i* can be written

(A13 )
$$c_{i}\;:\;=\frac{\sum_{k=1}^{n}\sum_{\ell =1}^{n}[S_{i}^{\left(k,\ell \right)}-D_{i}^{\left(k,\ell \right)}]}{\sum_{k=1}^{n}\sum_{\ell =1}^{n}[1-D_{i}^{\left(k,\ell \right)}]}=\frac{s_{i}-d_{i}}{1-d_{i}},$$

where we have used Equations (A8) and (A12).

If $c_{i}$ were observable, the probability of IBS sharing under an nIBD-to-IBS model would be approximated by it, with analogous reasoning to Appendix A.2.1.1: in the infinite sample size limit, that is, as *n* approaches infinity, $c_{i}$ would be expected to converge to the true conditional probability of IBS given nIBD (at least in the context of the standard law of large numbers). As such, we purport

(A14 )
$$P_{\mathrm{corrected}}(S_{i}^{\left(k,\ell \right)}=1\;|\;D_{i}^{\left(k,\ell \right)}=0)\approx c_{i}.$$

This construction allows us to capitulate the misspecification inherent in the standard nIBD-to-IBS model. In particular, rearranging Equation (A13), we see that the sample proportion of IBS pairs $s_{i}$—the standard nIBD-to-IBS model likelihood—can be written as a function of the unobservable proportion of IBD pairs $d_{i}$ and the unobservable sample proportion of nIBD individuals that are IBS $c_{i}$ as follows:

(A15 )
$$\text{Standard nIBD-to-IBS model}=s_{i}=c_{i}+(1-c_{i})d_{i}\neq c_{i}.$$

Otherwise stated, $d_{i}$ partially encodes relatedness structure in the sample proportion of IBS pairs $s_{i}$ and thus the standard nIBD-to-IBS model.

Since pairwise comparisons are performed with replacement, i.e. self–self comparisons are included, it is necessarily the case that $d_{i}\geq 1/n$, whereby $s_{i}\geq c_{i}$. As such, the standard nIBD-to-IBS model overestimates the probability of IBS for nIBD pairs at locus *i*. The degree of overestimation grows linearly as a function of the proportion of pairs $d_{i}$ that are IBD at locus *i*—and may therefore be particularly problematic in populations with high levels of relatedness.

***Aside***: Purcell *et al.* (2007) propose a correction for finite sample bias that is predicated on the probability that two identical alleles are selected from a pool of *n* alleles *without* replacement, i.e. excluding self–self comparisons. Implementing this correction yields the adjusted quantity

(A16 )
Adjusted nIBD-to-IBS model=si′:=1(n2)∑k=1n−1∑ℓ=k+1nSi(k,ℓ)=si−1n1−1n,

where we have substituted Equation (A12). For sufficiently large sample sizes *n*, both formulations $s_{i}\approx s_{i}^{\prime }$ are approximately equal. This adjustment is sufficient in an outbred setting where all pairs of distinct parasites are unrelated, but falls short for inbred parasite samples.

***Aside***: While we have adopted the convention of pairwise comparisons with replacement, in line with standard nIBD-to-observation models (Henden *et al.* 2018; Schaffner *et al.* 2018; Taylor, Jacob, *et al.* 2019), this decision does not affect the theoretical correction $c_{i}$. That is, if we define

di′:=1(n2)∑k=1n−1∑ℓ=k+1nDi(k,ℓ)

to be the proportion of distinct pairs that are IBD at locus *i*, then analogous reasoning yields the corrected nIBD-to-IBS model

(A17 )
$$c_{i}^{\prime }=\frac{\sum_{k=1}^{n-1}\sum_{\ell =k+1}^{n}[S_{i}^{\left(k,\ell \right)}-D_{i}^{\left(k,\ell \right)}]}{\sum_{k=1}^{n-1}\sum_{\ell =k+1}^{n}[1-D_{i}^{\left(k,\ell \right)}]}=\frac{s_{i}^{\prime }-d_{i}^{\prime }}{1-d_{i}^{\prime }}.$$

Inspection of Equations (A13) and (A17), however, reveals that $c_{i}=c_{i}^{\prime }$.

***Aside***: As for misspecification given IBD (Appendix A.2.1.1), besides in the unrealistic scenario where the parasite population is split into unrelated clonal clusters—in which case the finite-sample correction (A16) of Purcell *et al.* (2007) is directly applicable—removing replicates of clonal parasite from a parasite sample will not render $d_{i}$ equal zero, because individuals that are IBD at the *i*th locus are not necessarily IBD at all loci.

##### A.2.2 Consequences of the standard nIBD-to-IBS model under marker independence

The misspecification of the standard nIBD-to-IBS model leads to double-counting relatedness in the likelihood of IBS:

P(IBS)=P(IBD)+Pstandard(IBS|nIBD)⏞obs.model:inflatedbylocuswiseproportionIBD⋅[1−P(IBD)]⏟likelihoodofIBC=IBS∩nIBD;

intuitively, we expect this to lead to the systematic underestimation of pairwise relatedness (Case A, Box A2). To characterize more thoroughly the consequences of this misspecification, we restrict our attention to the loci-independence model of pairwise relatedness (Taylor, Jacob, *et al.* 2019) with IBS descriptives. For the parasite pair (k,ℓ), the likelihood of observing the sequence of IBS states

$$S^{\left(k,\ell \right)}=(S_{1}^{\left(k,\ell \right)},\ldots ,S_{m}^{\left(k,\ell \right)})$$

across a set of loci i=1,…m, conditional on pairwise relatedness $r^{\left(k,\ell \right)}$, is given by

(A18 )
$$\begin{array}{rl} & P_{\mathrm{standard}}(S^{\left(k,\ell \right)}\;|\;r^{\left(k,\ell \right)})\\ & =\prod_{i=1}^{m}(r^{\left(k,\ell \right)}+s_{i}(1-r^{\left(k,\ell \right)}){)}^{S_{i}^{\left(k,\ell \right)}}(1-r^{\left(k,\ell \right)}-s_{i}(1-r^{\left(k,\ell \right)}){)}^{1-S_{i}^{\left(k,\ell \right)}} \end{array}.$$

The results in the present section bear strong conceptual similarity to the work of Weir and Goudet (2017); however, we consider MLEs whilst Weir and Goudet (2017) addressed method of moments estimators of relatedness.

##### A.2.2.1 The pairwise relatedness parameter captures deviation from average relatedness

To aid reinterpretation by inspection, we rewrite the likelihood (A18) in the following form:

(A19 )
$$\begin{array}{rl} P_{\mathrm{standard}}(S^{\left(k,\ell \right)}\;|\;r^{\left(k,\ell \right)}) & =\prod_{i=1}^{m}[1-(1-s_{i})(1-r^{\left(k,\ell \right)}){]}^{S_{i}^{\left(k,\ell \right)}}[(1-s_{i})(1-r^{\left(k,\ell \right)}){]}^{1-S_{i}^{\left(k,\ell \right)}},\\ & =\prod_{i=1}^{m}\{[1-(1-c_{i})\{1-d_{i}-r^{\left(k,\ell \right)}(1-d_{i})\}{]}^{S_{i}^{\left(k,\ell \right)}}\\ & \;\;[(1-c_{i})\{1-d_{i}-r^{\left(k,\ell \right)}(1-d_{i})\}{]}^{1-S_{i}^{\left(k,\ell \right)}}\}, \end{array}$$

where $s_{i}=c_{i}+(1-c_{i})d_{i}$ (Equation (A15)) has been substituted into the first line.

Under the corrected nIBD-to-IBS model, $c_{i}$ (Equation (A13)), the probability of $S^{\left(k,\ell \right)}$ is analogously given by

(A20 )
$$\begin{array}{rl} P_{\mathrm{corrected}}(S^{\left(k,\ell \right)}\;|\;r_{\mathrm{corrected}}^{\left(k,\ell \right)}) & =\prod_{i=1}^{m}[1-(1-c_{i})(1-r_{\mathrm{corrected}}^{\left(k,\ell \right)}){]}^{S_{i}^{\left(k,\ell \right)}}\\ & \;\;\;\times [(1-c_{i})(1-r_{\mathrm{corrected}}^{\left(k,\ell \right)}){]}^{1-S_{i}^{\left(k,\ell \right)}} \end{array}.$$

From inspection of Equations (A19) and (A20), the probability of IBD sharing at locus *i* under the corrected model can be written in the form

( A21 )
Pcorrected(Di(k,ℓ)=1)=di⏟averagerelatednessinparasitesample+r(k,ℓ)(1−di)⏟deviationfromaveragerelatednessinparasitesample.

***Aside***: We do not equate $r_{\mathrm{corrected}}^{\left(k,\ell \right)}$ to $P_{\mathrm{corrected}}(D_{i}^{\left(k,\ell \right)}=1)$ because $d_{i}$ are not necessarily the same for all *i*.

We suggest that Equation (A21) captures the “true” likelihood of IBD sharing at locus *i*, and can be decomposed into two components:

1. the sample proportion of IBD pairs $d_{i}$ reflecting the average relatedness at locus *i* in the parasite sample; and
2. the term $r^{\left(k,\ell \right)}(1-d_{i})$ quantifying pairwise IBD sharing at locus *i* that can not be explained by the average locuswise relatedness in the parasite sample alone.

In practice, inference is performed on the parameter $r^{\left(k,\ell \right)}$. Rearranging Equation (A21), we can write

(A22 )
$$r^{\left(k,\ell \right)}=\frac{P_{\mathrm{corrected}}(D_{i}^{\left(k,\ell \right)}=1)-d_{i}}{1-d_{i}}.$$

Equation (A22) suggests that the parameter $r^{\left(k,\ell \right)}$ is intrinsically adjusted for locuswise average relatedness in the parasite sample, $d_{i}$, which varies across loci. In particular, we suggest that $r^{\left(k,\ell \right)}$ should be interpreted as a relative measure, quantifying how strongly relatedness between the parasite pair (k,ℓ) deviates from the locuswise average relatedness $d_{i}$ in the parasite sample. This mirrors the notion of relative relatedness articulated by Weir and Goudet (2017).

##### A.2.2.2 Relatedness estimates are stratified by average relatedness

In practice, the pairwise relatedness parameter $r^{\left(k,\ell \right)}$ is taken to have range [0,1]. From Equation (A21), however, we see that the constraint $r^{\left(k,\ell \right)}\geq 0$ introduces a lower bound

$$P_{\mathrm{corrected}}(D_{i}^{\left(k,\ell \right)}=1)\geq d_{i}$$

on the “corrected” model likelihood of IBD sharing at locus *i* for the parasite pair (k,ℓ). In particular, setting $r^{\left(k,\ell \right)}=0$ is equivalent to approximating the locuswise probability of pairwise IBD by the locuswise sample average relatedness $d_{i}$: plugging $r^{\left(k,\ell \right)}=0$ into Equation (A21) and evoking the assumed independence across markers, we obtain

$$P_{\mathrm{standard}}(D^{\left(k,\ell \right)}\;|\;r^{\left(k,\ell \right)}=0)=\prod_{i=1}^{m}d_{i}^{D_{i}}(1-d_{i}{)}^{1-D_{i}},$$

which can be interpreted as the expected distribution of pairwise relatedness for the parasite genotype sample. This observation echoes Weir and Goudet (2017), who additionally allude to the concept of negative relatedness.

The notion of ostensibly “unrelated” parasite pairs $r^{\left(k,\ell \right)}=0$ warrants attention. To explore this notion, it is instructive to consider an MLE scheme for the parameter $r_{k,\ell }$. From Equation (A19), we verify that the derivative of the log-likelihood for the sequence of observed IBS states $S^{\left(k,\ell \right)}$

(A23 )
$$\frac{d\log\;P_{\mathrm{standard}}(S^{\left(k,\ell \right)}\;|\;r^{\left(k,\ell \right)})}{dr^{\left(k,\ell \right)}}=\sum_{i=1}^{m}\left\{\frac{(1-s_{i})S_{i}^{\left(k,\ell \right)}}{s_{i}+(1-s_{i})r^{\left(k,\ell \right)}}-\frac{1-S_{i}^{\left(k,\ell \right)}}{1-r^{\left(k,\ell \right)}}\right\}$$

is a monotonically decreasing function of $r^{\left(k,\ell \right)}$. Equation (A23) allows us to identify a sufficient and necessary condition to recover a zero MLE ${\hat{r}}^{\left(k,\ell \right)}=0$:

(A24 )
$${\frac{d\log\;P_{\mathrm{standard}}(S^{\left(k,\ell \right)}\;|\;r^{\left(k,\ell \right)})}{dr^{\left(k,\ell \right)}}|}_{r^{\left(k,\ell \right)}=0}\leq 0\;⟺\;\frac{1}{m}\sum_{i=1}^{m}\frac{S_{i}^{\left(k,\ell \right)}}{s_{i}}\leq 1.$$

We now examine this threshold in greater detail. Recall from Equation (A15) that

$$s_{i}=d_{i}+(1-d_{i})c_{i},$$

where $c_{i}$ constitutes the corrected nIBD-to-IBS model. We can therefore interpret $s_{i}$ as the expected IBS sharing at locus *i*, predicated on the average relatedness $d_{i}$ at locus *i* in the parasite sample. As such, the observable threshold

(A25 )
$$\frac{1}{m}\sum_{i=1}^{m}\frac{S_{i}^{\left(k,\ell \right)}}{s_{i}}=1$$

can be thought to stratify pairs with below-average vs above-average relatedness. We interpret the LHS of Equation (A25) as a weighted measure of pairwise IBS sharing, with pairwise IBS at loci with a limited sample proportion of IBS pairs (i.e. small $s_{i}$) weighted more heavily.

Our interpretation of the MLE ${\hat{r}}^{\left(k,\ell \right)}$, mirroring Hall *et al.* (2012) and Weir and Goudet (2017), is thus two-fold:

- ${\hat{r}}^{\left(k,\ell \right)}=0$ indicates that the parasite pair (k,ℓ) has below-average relatedness relative to the parasite sample, but is otherwise uninformative (i.e. does not quantify the extent to which pairwise relatedness is below the average relatedness in the parasite sample). Permitting negative relatedness estimates, which arise naturally using moment estimators, may be informative for parasite pairs with below-average relatedness relative to the parasite sample (Hall *et al.* 2012).
- ${\hat{r}}^{\left(k,\ell \right)}>0$ quantifies the extent to which relatedness between the parasite pair (k,ℓ) exceeds the average relatedness in the parasite sample, or the amount of relatedness between the parasite pair (k,ℓ) that cannot be explained by average relatedness alone.

The adjustment for “average relatedness” in the parasite sample is predicated on the locuswise sample proportion of IBD pairs $d_{i}$. If $d_{i}$ is taken to be constant across all loci i=1,…,m, then from Equations (A20) and (A22), a *nonzero* MLE ${\hat{r}}^{\left(k,\ell \right)}$ can be written in the form

$${\hat{r}}^{\left(k,\ell \right)}=\frac{{\hat{r}}_{\mathrm{corrected}}^{\left(k,\ell \right)}-d}{1-d},$$

where ${\hat{r}}_{\mathrm{corrected}}^{\left(k,\ell \right)}$ is the MLE obtained under the corrected nIBD-to-IBS model $c_{i}$ and the index *i* is dropped on account of all $d_{i}$ being equal. A schematic of the relationship between ${\hat{r}}_{\mathrm{corrected}}^{\left(k,\ell \right)}$ and ${\hat{r}}^{\left(k,\ell \right)}$ is shown in Fig. A4. Accounting for the variability of $d_{i}$ across loci, we predict, would yield a fuzzy elbow-like characteristic, with a change point near the average sample relatedness

$$\bar{d}=\frac{1}{m}\sum_{i=1}^{m}d_{i}.$$

For a sample of individuals with a unimodal positively skewed distribution of pairwise relatedness, we would expect the majority of parasite pairs to exhibit “below-average” relatedness, manifest in pronounced zero inflation of the pairwise MLEs ${\hat{r}}^{\left(k,\ell \right)}$.

#### A.3 Numerical results

Here, we present various numerical results that complement our theoretical findings (Appendices A.3.1 and A.3.2). We additionally show that exploiting linkage structure within dense data using the HMM of relatedness mitigates underestimation driven by standard observation models (Appendix A.3.3). Zero-inflation in relatedness estimates under (n)IBD independence, arising from an over-reliance on the underlying (n)IBD-to-observational model, can be explained through comparative plots of IBS sharing (Appendix A.3.4). Finally, we propose a diagnostic to gauge the average locuswise relatedness $\bar{d}$ in a sample and approximate the severity of underestimation (Appendix A.3.5).

##### A.3.1 Relatedness is systematically underestimated using standard models

Relatedness is systematically underestimated using standard models (Fig. A5). This holds whether observations are IBS descriptives (plots C1, D1) or alleles (plots C2–C3, D2–D3), whether the standard model assumes (n)IBD independence (plots C1–C2, D1–D2) or not (plots C3, D3), and whether markers are dense or sparse (plots C and D, respectively). Estimates are severely zero-inflated when data are sparse (plots B2–B4) and when dense data are fit under (n)IBD independence (plots A2–A3). Less severe zero-inflation and underestimation when the HMM is fit to dense data (plots A4 and C3, respectively) is addressed in Appendices A.3.3 and A.3.4. Zero inflation renders $\text{mean}(\hat{r})$ under (n)IBD independence a poor approximation of $\;\bar{\;d}$.

##### A.3.2 Underestimation using standard models is due to the partial encoding of population relatedness in sample allele frequencies

The degree of underestimation of pairwise relatedness using standard models increases with the number of generations of inbreeding because the standard nIBD-to-nIBS model is increasingly misspecified, in that the sums of squares of sample allele frequencies encode more relatedness (Fig. A6). The unbiased nature of relatedness estimates generated under the corrected independence model fit to (n)IBS observations (Fig. A7) also supports, by contrast, the idea that underestimation is due to the use of sample allele frequencies within standard observation models. This is true of both dense and sparse data, although estimates based on sparse data are less precise.

##### A.3.3 Relatedness is less severely underestimated using the standard HMM fit to dense data

Consider an inbred population with elevated relatedness (Fig. A8a). Increasing the marker count while accounting for linkage (estimation under the HMM with the standard (n)IBD-to-allele model) generates more precise and less biased estimates (Fig. A8b). Meanwhile, increasing the marker count without accounting for linkage (estimation under (n)IBD independence with either the standard nIBD-to-IBS or (n)IBD-to-allele model) does not remove bias (Fig. A8c, d). We believe this is because estimation under the HMM exploits linkage information, which increases with marker density. Meanwhile, estimation under the independence model does not exploit linkage information, regardless of its extent in the data. Instead, the independence model is entirely reliant on sample allele frequencies, which render the observational model misspecified. Otherwise stated, the standard HMM is less reliant upon sample allele frequencies and thus less susceptible to the misspecification they cause.

***Aside***: Under the HMM, the extent to which pairwise relatedness is underestimated decreases over the zero to one range granted data are sufficiently dense to encode linkage structure (Fig. A9). This is because, for a given marker count, the extent of linkage depends on the length of shared IBD segments, which is greater for recent relatives, and thus correlated with relatedness. We mention this as an aside only, because linkage due to recent ancestry is an inherent property of a given parasite genotype pair, not something we can control.

##### A.3.4 Comparative plots of IBS distributions capture the extent of zero-inflation under the independence model

The persistence of zero inflation in dense data relatedness estimates under the independence model can be explained through comparative plots of IBS distributions.

For a given pair of individuals, the fraction of polymorphic sites that are IBS can be computed. The computation can then be repeated for all possible pairs. Hereafter, we refer to this set of values as the empirical IBS distribution for all pairs. As a comparator, under the standard nIBD-to-IBS model, i.e. $s=(s_{1},\ldots ,s_{n_{\mathrm{markers}}})$, the expected IBS distribution for ostensibly unrelated parasite pairs (r=0) comprises a rescaled Poisson binomial distribution (i.e. a sum of independent, but not necessarily identically distributed Bernoulli random variables):

$$Y_{0}(s)=\frac{1}{n_{\mathrm{markers}}}\sum_{i=1}^{n_{\mathrm{markers}}}X_{i}(s_{i})$$

where

$$X_{i}(s_{i})\overset{\mathrm{independent}}{∼}\;\text{Bernoulli}(s_{i}).$$

$Y_{0}(s)$ is also applicable under the HMM, because the HMM collapses down to the independence model for unrelated pairs.

Comparison of the empirical IBS distribution for all pairs and the expected IBS distribution for ostensibly unrelated pairs (Fig. A10a) shows that there is a range of empirical IBS values less than the expected IBS distribution for ostensibly unrelated pairs. The majority of these pairs have zero-valued relatedness estimates under the independence model, because the independence model is heavily reliant on the standard nIBD-to-IBS model (Fig. A10b, orange). Otherwise stated, the extent to which empirical IBS values fall below the expected IBS distribution for unrelated pairs corresponds to the extent of zero-inflation under independence. This result is consistent with the notion that locuswise average relatedness is partially encoded within the locuswise sample proportion of IBS pairs $s_{i}$, whereby the expected IBS distribution for ostensibly unrelated pairs ought to be reinterpreted as the expected IBS distribution for pairs with “average” relatedness. Consequently, a range of below-average relatedness values map onto zero-valued relatedness estimates under the independence model. Meanwhile, they have small but nonzero estimates under the HMM model, because the HMM is less reliant on the underlying (n)IBD-to-observation model (Fig. A10b, green).

##### A.3.5 Dense-dense data plots approximately capture the extent of underestimation

We leverage the precision of estimates generated using dense data to design a practical diagnostic for identifying approximately the corrective value $\bar{d}$. More specifically, although relatedness estimates generated under the HMM using dense marker data from inbred populations are underestimates (Figs. A8 and A9), they are sufficiently less biased downwards compared with those generated under (n)IBD independence to reveal an elbow-like pattern (Fig. A11b), which is otherwise only accessible using either simulation (Fig. A11a) or theory (Fig. A4).

#### A.4 Empirical results

We devote Appendix A.4 to the empirical effect of population structure, under which our theoretical and numerical results do not necessarily hold. To do so, we pool the n=278 isolates from Guyana (Vanhove *et al.* 2024), analyzed in the main text, with an additional n=28 isolates from Colombia (Carrasquilla *et al.* 2022), yielding n=30,694 biallelic SNPs that are polymorphic among the pooled isolates. Population structure appears pronounced: principal coordinates analysis (PCoA), based on pairwise fractions of IBS markers, yields two distinct clusters, stratified by country (Fig. A12).

##### A.4.1 Additional manifestations of population structure

We examine two additional plots that indicate population structure whilst linking directly with our conceptual framework. First we examine the empirical distribution of pairwise fractions of IBS markers. In the absence of population structure, we would expect to see a unimodal IBS distribution. The multimodal IBS distribution shown in Fig. A13, in contrast, is indicative of population structure (Plucinski and Barratt 2021): we view it as a mixture distribution, with components stratified by within- and between-subpopulation comparisons. The well-separated components suggest pronounced differentiation between allele frequencies in the constituent subpopulations.

An alternative approach, linking back to our characterization of observation models predicated on allelic states vs IBS descriptives in Appendix A.2.1, is the comparison of relatedness estimates under (n)IBD independence predicated on the standard (n)IBD-to-allele model vs the standard nIBD-to-IBS model (Fig. A14). Allelic states are more informative than IBS descriptives. Under the (n)IBD-to-allele model, a shared minor allele points towards IBD more strongly than a shared major allele; this relative weighting is erased when we shift to IBS descriptives. In the absence of population structure, we would expect each pair of individuals to share a mixture of major and minor alleles; a shift from allelic states to IBS descriptives would therefore introduce noise, but we would expect the over/under-weighting of shared major/minor alleles to average out across markers. We would, however, expect systematic differences to emerge in the presence of population structure. Comparisons within the major subpopulation, for instance, systematically yield parasite pairs with a shared major allele, yielding higher relatedness estimates based on IBS descriptives relative to allelic states; the converse applies to the minor subpopulation. Systematic patterns akin to those in Fig. A14 are thus indicative of population structure.

##### A.4.2 Dense data diagnostic in the presence of population structure

We now examine the dense data diagnostic proposed in Appendix A.3.5 in the presence of population structure. In the absence of population structure (main text), we observe a clean elbow-like pattern concordant with our theoretical predictions (Fig. A4) and numerical results (Fig. A11). Population structure, however, yields the emergence of multiple elbows, corresponding to different within- and between-population comparisons, with a different structure depending on whether estimates under (n)IBD independence are predicated on allelic states or IBS descriptives (Fig. A15). Because sample allele frequencies represent a weighted average across subpopulations, interpretation of the branch points is unclear. The apparent absence of bias using allelic states for the inbred minor subpopulation (Colombia) is unclear given this weighted average of sample allele frequencies. However, comparison of estimates using allelic states vs IBS descriptives under (n)IBD independence (Fig. A14) suggests that nonrepresentative sample allele frequencies may introduce an upward bias in relatedness estimates in certain contexts: alleles that are common in the minor subpopulation, but uncommon in the major subpopulation, may inflate relatedness estimates within the minor subpopulation.

##### A.4.3 Zero inflation and population structure

The standard nIBD-to-IBS model leads to the systematic over/under-weighting of shared major/minor alleles relative to the standard (n)IBD-to-allele model. When sample allele frequencies are averaged over several subpopulations and estimates are generated under (n)IBD independence, allelic states yield zero inflation in comparisons within the major subpopulation; while IBS descriptives yield zero inflation in comparisons within the minor subpopulation (Fig. A16). Theoretically predicted and numerically validated results pertaining to zero inflation, therefore, are sensitive to population structure. Empirically, variable zero inflation under (n)IBD independence and the nIBD-to-IBS model for within- and between-population comparisons can be directly explained by the multimodal distribution of pairwise IBS sharing (Fig. A13) but does not have a direct interpretation.

### Appendix B: Simulation model

We describe a simulation model that starts with a population whose ancestry is defined in terms of a bygone outbred founder population and then simulates successive generations of inbreeding. Our model differs from the canonical Wright–Fisher model of stochastic drift, which concerns a single locus (Etheridge 2011), on three grounds:

- We simulate multiple loci and introduce linkage between them through a discrete-time homogeneous Markov chain governing crosses.
- We introduce clonal and sibling substructure through relationship graphs (Taylor, Watson, *et al.* 2019).
- On top of the sibling and clonal substructure, we introduce an increased propensity for selfing and sibling–sibling mating through a single parameter; in doing so, we capture departure from panmixia. In reality, a single parameter cannot represent multiple processes that govern selfing and sibling–sibling mating between malaria parasites: selfing between malaria parasites is always viable theoretically; given a monoclonal mosquito infection, it is inevitable. Mosquito-to-human-to-mosquito cotransmission enhances the probability of sibling–sibling mating (Camponovo *et al.* 2022).

Demographic processes like immigration, mutation, and selection are not accounted for.

Our description is structured as follows. In Appendix B.1, we introduce a model under which offspring are generated as a mosaic of two parents, allowing for linkage between markers which are all equidistant. We then propose a model for our “generation zero” population, whose ancestry is formulated in terms of a bygone outbred founder population (Appendix B.2). In Appendix B.3, we propose a model under which the population in the present generation guides the generation of the population in the next generation. Designed to capture a single generation of inbreeding, our framework is underpinned by the relationship graph model of Taylor, Watson, *et al.* (2019). In Appendix B.4, we detail our approach for assessing misspecification under the standard nIBD-to-IBS model. A summary of simulation parameters, and their respective tradeoffs, is provided in Appendix B.5.

#### B.1 Generating a single individual with inter-marker linkage

We consider some number, $n_{\mathrm{markers}}$, of multiallelic, *equidistant* markers, spanning a single chromosome, and indexed $i=1,\ldots ,n_{\mathrm{markers}}$, with each marker treated as a point polymorphism (Taylor, Jacob, *et al.* 2019). Each time we simulate a single meiosis, we generate a single offspring as a random $n_{\mathrm{markers}}$-mosaic of parents that are labeled $\left\{a_{1},a_{2}\right\}$, respectively. In reality, a single meiosis between two parental genotypes generates four meiotic offspring, which then replicate asexually into thousands of sporozoites; on average, meiotic offspring are related to each other by 1/3 and to each parent by 1/2 if parental genotypes are unrelated (Wong *et al.* 2018; Taylor, Watson, *et al.* 2019).

Denote by

$$b=(b_{1},\ldots ,b_{n_{\mathrm{markers}}})$$

the mosaic of an offspring of parents $\left\{a_{1},a_{2}\right\}$, with $b_{i}=a_{1}$ if marker *i* is inherited from parent $a_{1}$ and $b_{i}=a_{2}$ otherwise.

Assuming a genomewide constant recombination rate of *ρ*, b is governed by a discrete-time, homogeneous Markov chain with transition matrix

$$\left(\begin{array}{cc} P(b_{i+1}=a_{1}\;|\;b_{i}=a_{1}) & P(b_{i+1}=a_{1}\;|\;b_{i}=a_{2})\\ P(b_{i+1}=a_{2}\;|\;b_{i}=a_{1}) & P(b_{i+1}=a_{2}\;|\;b_{i}=a_{2}) \end{array}\right)=\left(\begin{array}{cc} 0.5(1+e^{-\rho d}) & 0.5(1-e^{-\rho d})\\ 0.5(1-e^{-\rho d}) & 0.5(1+e^{-\rho d}) \end{array}\right),$$

equivalent to the Markov model of relatedness (Equation (A3)) in the case r=0.5 with switch rate κ=1 where *d* is measured in base pairs (bp) and the recombination rate *ρ* has units M/bp.

Due to discrete sampling of the genome, consecutive markers inherited from the same parent may be separated by an even number of recombination breakpoints, which occur between base pairs. For the purposes of simulating ancestry at a fixed set of markers, we do not distinguish whether or not a string of markers inherited from the same parent is separated by recombination breakpoints (Fig. B1), and assume that a Poisson process governs the position of recombination breakpoints (Speed 1997).

Observe that

$$\begin{array}{rl} P(b_{i+1}=b_{i+2} & =\ldots b_{i+m-1}=a_{1},b_{i+m}=a_{2}\;|\;b_{i}=a_{1})\\ & =\frac{1}{2^{m}}(1-e^{-\rho d})(1+e^{-\rho d}{)}^{m-1} \end{array}.$$

If we ignore (temporarily) the finite number of markers $n_{\mathrm{markers}}$, then given $b_{i}=a_{1}$, the number of consecutive markers (from *i* inclusive) inherited from parent $a_{1}$ is geometrically distributed with state space N and mean length

(B1 )
$$\bar{M}\;:\;=\frac{2}{1-e^{-\rho d}}.$$

Stretches of consecutive markers inherited from parent $a_{2}$ are identically distributed in length.

We can therefore treat b as a truncated alternating renewal process, where segments alternate from each parent and are independent and identically distributed (i.i.d.) with length

$$L∼\text{Geometric}\left(\frac{1}{\bar{M}}\right),$$

where the geometric distribution is taken to have support N. The case $\bar{M}=2$ reduces to the independence model.

To simulate an offspring under this model, we sample a parent $a_{1}$ or $a_{2}$ with probability 1/2; sample a geometric segment length; select the alternative parent and re-sample a geometric segment length; string together alternating geometric segments from each parent; and then truncate once a length of $n_{\mathrm{markers}}$ has been obtained, to account for the finite length that is ignored above.

#### B.2 Generating a population of individuals at generation zero

We now propose a model under which to generate a “generation zero” population whose ancestry is constructed in terms of a bygone outbred founder population. This construction allows us to distribute background relatedness across the generation zero population.

Firstly, let us consider a founder population comprising some number of unrelated founders, $n_{\mathrm{founders}}$. At each marker $i=1,\ldots ,n_{\mathrm{markers}}$, we select the allele q∈{1,…,y} with probability $p_{i}(q)$

$$A_{\mathrm{founder}}(f,i)\overset{i.i.d.}{∼}\;\text{Categorical}(p_{i}(1),\ldots ,p_{i}(y))$$

independently for each founder $f=1,\ldots ,n_{\mathrm{founders}}$. Here, multiallelic markers are treated as point polymorphisms (Taylor, Jacob, *et al.* 2019). For large $n_{\mathrm{markers}}$, each founder is likely to harbor a unique allelic sequence.

We represent individuals within subsequent generations as mosaics of the $n_{\mathrm{founders}}$ founders; that is the ancestry of individual *j* in generation *k* is represented in the form

$$F(j,k)=(f_{1},\ldots ,f_{n_{\mathrm{markers}}})\in \{1,\ldots ,n_{\mathrm{founders}}{\}}^{n_{\mathrm{markers}}}.$$

Barring genotyping error and mutations, the vector of allelic states observed for individual *j* in generation *k* is then given by

$$A(j,k)=\left(A_{\mathrm{founder}}(f_{1},1)\ldots ,A_{\mathrm{founder}}(f_{n_{\mathrm{markers}}},n_{\mathrm{markers}})\right).$$

The ancestry of each individual *j* in generation zero is independently sampled uniformly at random from the set of all founder sequences, that is,

(B2 )
$$F(j,0)\overset{i.i.d.}{∼}\;\text{Uniform}[\{1,,\ldots ,n_{\mathrm{founders}}{\}}^{n_{\mathrm{markers}}}],$$

and we initialize a population relationship graph for generation zero by placing stranger edges between all individuals/nodes.

Alleles that are derived from the same founder are designated IBD. Alleles that are IBS but not derived from the same founder are designated IBC. Given parameter values listed in Table B1, the vast majority of IBS in generation zero is due to IBC and not IBD. We call this low-level IBD in generation zero background relatedness.

**Table B1.** Summary of simulation parameters.

Feature	Parameter	Interpretation	Value
Markers	$n_{\mathrm{markers}}$	Number of equidistant markers distributed across a single chromosome	24,000
	ρd	Distance between consecutive markers in Morgans	≈0.002
	$\bar{M}=2(1-e^{-\rho d}{)}^{-1}$	Average number of consecutive markers inherited from the same parent in a cross (reduces to $\bar{M}=2$ given locus independence)	1,000
Relationship graphs	$n_{\mathrm{individuals}}$	Number of not necessarily distinct genotypes, each representing an equal number of actual parasites	100
	$m_{\mathrm{subgraph}}$	Size of transitive clone/sibling/stranger graphs (Taylor, Watson, *et al.* 2019) sampled uniformly at random; interpretable as a multiplicity of infection upper bound (if subgraphs are viewed as parasite genotypes within a host) or the number of successful, not necessarily distinct genotypes per bite (if subgraphs are viewed as successfully transmitted sporozoites per bite)	4
Founder	$n_{\mathrm{founders}}$	Number of unrelated founders	100
	*y*	Number of possible alleles at each at marker	2
	$p_{i}(q)$	Probability of each founder *f* harboring allele q∈{1,…,y} at marker *i*	(0.1,0.9)
Inbreeding	$n_{\mathrm{generations}}$	Number of discrete, nonoverlapping generations of inbreeding [exc. 0]	10
	$p_{\mathrm{cotransmission}}$	Probability of sampling parents without replacement from the same constituent subgraph *vs* with replacement from the population as a whole in the previous generation for each sibling/clonal component in a relationship graph	0.4

Under this model, low-level background relatedness is maximally spread across parasite pairs in generation zero. Implicit in this construction is the assumption that there is sufficient temporal separation between the founder population and generation zero to break down inter-marker linkage. If we instead modeled generation zero as one generation of mating from the founder population, relatedness would be concentrated on a small subset of pairs with shared parents, consistent with recent rather than background relatedness (Appendix B.5.1), and Equation (B2) would not hold because each individual in generation zero would be constructed as a mosaic of at most two founders.

***Aside***: The uniform assumption (Equation (B2)) is also compatible with an (n)IBD-to-allele model predicated on founder allele frequencies. Because Equation (B2) holds, the probability of randomly sampling the allelic sequence

$$A=(q_{1},\ldots ,q_{n_{\mathrm{markers}}})$$

for each individual in generation zero is proportional to the number of ways of recovering that sequence as a mosaic of founders:

(B3 )
$$\begin{array}{r} P(A)=\frac{1}{(n_{\mathrm{founders}}{)}^{n_{\mathrm{markers}}}}\sum_{\;f_{1}=1}^{n_{\mathrm{founders}}}\ldots \sum_{\;f_{n_{\mathrm{markers}}}=1}^{n_{\mathrm{founders}}}\prod_{i=1}^{n_{\mathrm{markers}}}1\{A_{\mathrm{founder}}(f_{i},i)=q_{i}\} \end{array}.$$

Interchanging the summation and product in Equation (B3), we can equivalently write

(B4 )
$$\begin{array}{rl} P(A) & =\frac{1}{(n_{\mathrm{founders}}{)}^{n_{\mathrm{markers}}}}\prod_{i=1}^{n_{\mathrm{markers}}}\sum_{\;f=1}^{n_{\mathrm{founders}}}1\{A_{\mathrm{founder}}(f,i)=q_{i}\}\\ & =\prod_{i=1}^{n_{\mathrm{markers}}}\theta_{\mathrm{founder}}(q,i), \end{array}$$

since the frequency of allele *q* at locus *i* in the founder population is

(B5 )
$$\theta_{\mathrm{founder}}(q,i)\;:\;=\frac{1}{n_{\mathrm{founders}}}\sum_{\;f=1}^{n_{\mathrm{founders}}}1\{A_{\mathrm{founder}}(f,i)=q\}.$$

Equation (B4) is precisely the likelihood of observing the allelic sequence A that we would obtain by plugging founder allele frequencies (B5) into the standard nIBD-to-allele model under locus independence. In other words, our construction gives rise to an allelic distribution in generation zero that is identical to that which we would obtain by directly drawing alleles with founder allele frequencies, but with the added advantaging of enabling us to explicitly define low-level background relatedness across parasite pairs in generation zero.

#### B.3 Generating populations of individuals over successive generations of inbreeding

From generation k=1 onwards, we simulate successive discrete, nonoverlapping generations of inbreeding. We capture stochastic drift, with the imposition of additional sibling/clonal substructure in the line with the formulation of Taylor, Watson, *et al.* (2019); but ignore the phenomena of immigration, mutation, and selection.

We assume that the population size $n_{\mathrm{individuals}}$ remains fixed across generations. As stated above, the population relationship graph for generation zero has stranger edges between all individuals. To simulate the ancestry of generation k≥1, given that of generation (k−1), we perform the following steps:

1. Simulate a population-level relationship graph for generation *k*, characterized by clonal, sibling, and stranger edges (Appendix B.3.1).
2. Conditional on the structure of the relationship graphs for generations (k−1) and *k*, formulate each individual in generation *k* as a mosaic of parents from generation (k−1), allowing for an enriched probability of sibling–sibling crosses and selfing over generations (Appendix B.3.1.1).
3. Recover the ancestry structure (relative to founders $f=1,\ldots ,n_{\mathrm{founders}}$) for each individual in generation *k* using the encoding F(j,k−1).

The simulation structure is informed by the work of Taylor, Watson, *et al.* (2019), and draws on the R package Pv3Rs (Taylor 2022b). Below, we describe the first two steps in detail.

##### B.3.1 Generating relationship graphs

For each generation k≥1, we simulate a transitive relationship graph with sibling, clonal, and stranger edges, following the framework of Taylor, Watson, *et al.* (2019). Relationship graphs, which are designed to capture one generation of inbreeding, are sampled independently for each generation. These graphs are fully connected, in that each pair of nodes is necessarily connected by either a sibling, clonal, or stranger edge; but transitivity is enforced for sibling and clonal edges.

We generate each population-level relationship graph, comprising $n_{\mathrm{individuals}}$ nodes, by amalgamating subgraphs of fixed size $m_{\mathrm{subgraph}}$ as follows. We first simulate $n_{\mathrm{individuals}}/m_{\mathrm{subgraph}}$ constituent subgraphs uniformly at random over the space of transitive graphs with $m_{\mathrm{subgraph}}$ labeled nodes and (undirected) sibling/clonal edges. The transitive property dictates that if nodes *x* and *y*, and nodes *y* and *z* are each connected by a clonal (sibling) edge, then nodes *x* and *z* must also be connected by a clonal (sibling or clonal) edge. For example, in the case $m_{\mathrm{subgraph}}=4$, we can construct 60 possible labeled transitive graphs with sibling/clonal/stranger edges. Removing node labels yields 14 unique configurations, as shown in Fig. B2. Sampling uniformly at random over the 60 possible labeled subgraphs yields the distribution shown in Fig. B2 over the 14 unique graph configurations. Each subgraph is sampled independently using the function sample_RG, implemented in the R package Pv3Rs (Taylor 2022b). To amalgamate constitutent subgraphs, we ascribe stranger edges for all between-subgraph comparisons.

As a consequence of this construction, which is motivated in part by computational constraints, population-level relationship graphs (for $n_{\mathrm{individuals}}$) are sampled over a nonuniform distribution across the space of transitive graphs with $n_{\mathrm{individuals}}$ labeled vertices and (undirected) sibling/clonal/stranger edges, that is biased towards those containing small, relatively balanced subclusters of clones and siblings: while each subgraph is of fixed size $m_{\mathrm{subgraph}}$, each clonal/sibling component is at most of size $m_{\mathrm{subgraph}}$. When subclusters are balanced, the assumption of conditional independence between allelic and IBD states that underpins the allelic observation model is justified (Fig. A2, Appendix A.2.1.1).

We view simulated individuals within subgraphs as successfully transmitted sporozoites per mosquito bite, and $m_{\mathrm{subgraph}}$ as the sporozoite count per bite. Of those sporozoites, all could be clonal, as in a monoclonal inoculation; all could be siblings, as in a multiclonal inoculation from a mosquito in which two genetically distinct parasites recombined, etc. The frequencies of these different scenarios are governed by the distribution over transitive graphs (Fig. B2a), which is uniform for convenience. Cotransmission is modeled through subgraphs derived by sampling parents from a previous subgraph, as captured below through the parameter $p_{\mathrm{cotransmission}}$. Superinfection, which implies independent bites, is modeled through subgraphs derived by sampling parents from the population at large. However, under this construction, bites (and thus genotypes) are not conceptually allocated to human hosts. An additional model is required to allocate bites to humans; it is superfluous for our purposes, but may be relevant for other applications.

##### B.3.1.1 Generating ancestry and thus genotypes conditional on relationship graphs for the present and previous generation

Given relationship graphs for generations (k−1) and *k*, we use Algorithm 1 to simulate the ancestry structure of generation *k* relative to generation (k−1); that is, we formulate individuals in generation *k* as crosses between parents from generation (k−1). Each sibling/clonal component in generation *k* is independently assigned two parents from generation (k−1). Siblings in generation *k* are modeled as *independent* crosses of the same parental pair from generation (k−1), and therefore have expected relatedness 1/2 (Wong *et al.* 2018; Taylor, Watson, *et al.* 2019). Clones in generation *k* are modeled as identical crosses of a given parental pair from generation (k−1).

For each sibling/clonal component in the relationship graph for generation *k*, parental pairs are selected under two possible schemes:

- With probability $p_{\mathrm{cotransmission}}$, we sample a constituent subgraph of size $m_{\mathrm{subgraph}}$ (that is, one of the $n_{\mathrm{individuals}}/m_{\mathrm{subgraph}}$ subgraphs we generated uniformly at random over the set of transitive relationship graphs of size $m_{\mathrm{subgraph}}$) uniformly at random in generation (k−1); and then select two parents uniformly at random *without* replacement from that constituent subgraph, yielding an elevated probability of sibling–sibling crosses and selfing. While parents are guaranteed to be sampled from the same subgraph, inbreeding is not guaranteed because a subgraph can contain strangers (Fig. B2a).
- With probability $\left(1-p_{\mathrm{cotransmission}}\right)$, we sample two parents from generation (k−1) uniformly at random *with* replacement, whereby parentals may be derived from the same subgraph but are more likely from different subgraphs.

This sampling scheme is designed to capture stochastic drift, with the enriched probability of selfing and sibling–sibling crosses acting as a proxy for monoclonal mosquito infections and serial cotransmission (Wong *et al.* 2018).

**Algorithm 1:** Simulate one generation of recombination (based on function sample_lineages of R package Pv3Rs  Taylor 2022b)

**Input:** transitive relationship graph $G_{\mathrm{current}}$ for current generation with sibling/clonal/stranger edges possible parents $\ell =1,\ldots ,n_{\mathrm{parents}}$ stratification of parents $L_{\mathrm{parent}}(g)\subset \{1,\ldots ,n_{\mathrm{parents}}\}$ by parental subgraph $g\in \{1,\ldots ,n_{\mathrm{individuals}}/m_{\mathrm{subgraph}}\}$ probability of cotransmission $p_{\mathrm{cotransmission}}$ number of $n_{\mathrm{markers}}$ at which individuals are genotyped size of parasite population $n_{\mathrm{individuals}}$ in subsequent generation
**Result** ancestry matrix $M\in \{1,\ldots ,n_{\mathrm{parents}}{\}}^{n_{\mathrm{individuals}}\times n_{\mathrm{markers}}}$ for subsequent generation
1 Delete all stranger edges in $G_{\mathrm{current}}$;
2 Decompose $G_{\mathrm{current}}$ into fully-connected sibling/clonal subgraphs $S_{1},\ldots ,S_{n_{\mathrm{subgraph}}}$;
3 **for** $j\in \{1,\ldots ,n_{\mathrm{subgraph}}\}$ **do**
4 Delete all sibling edges in $S_{j}$;
5 Decompose $S_{j}$ into clonal components $C_{1}^{j},\ldots ,C_{s_{j}}^{j}$;
6 **if** $u∼U(0,1)<p_{\mathrm{cotransmission}}$ **then**
7 Sample parental subgraph $g\in \{1,\ldots ,n_{\mathrm{individuals}}/m_{\mathrm{subgraph}}\}$ uniformly at random;
8 Sample $\ell_{1},\ell_{2}\in L_{\mathrm{parent}}(g)$ uniformly at random, without replacement;
9 **else**
10 Sample $\ell_{1},\ell_{2}\in \{1,\ldots ,n_{\mathrm{parents}}\}$ uniformly at random, with replacement;
11 **for** $C_{s}^{j}\in \{C_{1}^{j},\ldots ,C_{s_{j}}^{j}\}$ **do**
12 Simulate one meiosis $\ell_{\mathrm{offspring}}∼\text{Meiosis}(\ell_{1},\ell_{2})$ between $\ell_{1},\ell_{2}$
13 **for** *individuals i in clonal component* $C_{s}^{j}$ **do**
14 $M[i,\;]\leftarrow \ell_{\mathrm{offspring}}$
15 **return** M

#### B.4 Simulation model application

We treat the realized fraction of polymorphic markers that are IBD

$$R(j_{1},j_{2},k)\;:\;=\frac{\sum_{i=1}^{n_{\mathrm{markers}}}1\left\{F_{i}(j_{1},k)=F_{i}(j_{2},k)\right\}\cdot 1\{|\cup_{\;j=1}^{n_{\mathrm{individuals}}}A_{i}(j,k)|>1\}}{\sum_{i=1}^{n_{\mathrm{markers}}}1\{|\cup_{\;j=1}^{n_{\mathrm{individuals}}}A_{i}(j,k)|>1\}}$$

as the truth value for the pairwise relatedness of individuals $j_{1},j_{2}$ in generation *k*.

To diagnose systematic biases in relatedness estimation, we then generate MLEs of the pairwise relatedness parameter *r* and, where applicable, the switch rate parameter *κ*, under both the independence model of relatedness and the HMM using:

- the standard nIBD-to-IBS and (n)IBD-to-allele models predicated on sample allele frequencies;
- the corrected nIBD-to-IBS model based on the sample proportion of nIBD pairs that are IBS at each locus *i*, that is,
  $$c_{i}\;:\;=\frac{\sum_{1\leq j_{1}<j_{2}\leq n_{\mathrm{individuals}}}[1\left\{A_{i}(j_{1},k)=A_{i}(j_{2},k)\right\}-1\left\{F_{i}(j_{1},k)=F_{i}(j_{2},k)\right\}]}{\sum_{1\leq j_{1}<j_{2}\leq n_{\mathrm{individuals}}}[1-1\left\{F_{i}(j_{1},k)=F_{i}(j_{2},k)\right\}]},$$

based on polymorphic markers only. MLEs $\left(\hat{r},\hat{\kappa }\right)$ predicated on (n)IBD-to-allele models are generated using the R package paneljudge (Taylor 2022a), which implements both the HMM and independence model of relatedness. MLEs for the relatedness parameter *r* predicated on the nIBD-to-IBS model coupled with the (n)IBD independence model are computed using a custom R script. We do not generate estimates under an HMM with a nIBD-to-IBS observation model.

Under the ancestrally oblivious HMM of relatedness, each parameter set (r,κ) yields a distribution for the realized fraction of IBD markers (Speed and Balding 2015; Taylor, Jacob, *et al.* 2019). Convergence of the realized fraction of IBD markers to the relatedness parameter *r* occurs in the limit where an infinite number of equidistant markers are sampled along an infinitely long genome. While we acknowledge the conceptual difference between the pairwise relatedness parameter *r* and the realized fraction of sites IBD (Speed and Balding 2015; Taylor, Jacob, *et al.* 2019), the fact that we model recombination from ancestral principles means that, under our framework, there is no quantity with direct equivalence to *r*.

#### B.5 Summary of simulation parameters

We now examine the effects of simulation parameters on distributions of realized IBD/IBS sharing. A summary of simulation parameters is provided in Table B1.

##### B.5.1 Effect of $n_{\mathrm{individuals}}\;\text{vs}\;n_{\mathrm{founders}}$ on background relatedness

We distinguish two sources of relatedness within our simulation framework:

- “Recent” relatedness, attributed to crosses between shared parents from generation one onwards.
- “Background” relatedness, manifest in generation zero parasites, arising from the finite number of founders $n_{\mathrm{founders}}$.

These two sources of relatedness are different from identity by chance, which stems from limited marker cardinality under our simulation framework.

The population size, $n_{\mathrm{individuals}}$ and the number of founders $n_{\mathrm{founders}}$ govern the degree of background relatedness at generation zero. At locus *i*, each generation zero parasite is assigned ancestry from a founder $f\in \{1,\ldots ,n_{\mathrm{founders}}\}$, with independent assignment across both loci and parasites (Appendix B.2). The proportion of pairs IBD at locus *i* in generation zero, $d_{i}^{0}$, can thus be written

$$d_{i}^{0}=\frac{X_{1}^{2}+\ldots +X_{n_{\mathrm{founders}}}^{2}}{n_{\mathrm{individuals}}^{2}}$$

where

$$(X_{1},\ldots ,X_{n_{\mathrm{founders}}})∼\text{Multinomial}(n_{\mathrm{individuals}},(1/n_{\mathrm{founders}},\ldots ,1/n_{\mathrm{founders}})),$$

and pairs have been sampled with replacement.

In particular, the expected locuswise relatedness at generation zero is given by

$$E[d_{i}^{0}]=1-\left(1-\frac{1}{n_{\mathrm{individuals}}}\right)\left(1-\frac{1}{n_{\mathrm{founders}}}\right).$$

The degree of background relatedness exhibited by generation zero parasites influences realized IBD distributions in early generations that follow. It also governs the rate at which sibling and half-sibling pedigrees diverge from the expected relatedness 0.5 and 0.25, respectively. For the chosen parameter values $n_{\mathrm{founders}}=n_{\mathrm{individuals}}=100$ (Table B1), $E[d_{i}^{0}]\approx 2\%$.

##### B.5.2 Contribution of sibling/clonal subgraphs: $m_{\mathrm{subgraph}}$ $\text{ vs }n_{\mathrm{individuals}}$

The relative contributions of stranger/sibling/clonal edges in a population-level relationship graph—which are functions of $n_{\mathrm{individuals}}$ and $m_{\mathrm{subgraph}}$, respectively—are important determinants of relatedness. At each generation, we independently sample $n_{\mathrm{individuals}}/m_{\mathrm{subgraph}}$ subgraphs of size $m_{\mathrm{subgraph}}$ (uniformly at random, over the set of transitive graphs (Taylor, Watson, *et al.* 2019; Taylor 2022b)) and knit them together with stranger edges. As such, all between-subgraph comparisons—which constitute proportion $(n_{\mathrm{individuals}}-m_{\mathrm{subgraph}})/n_{\mathrm{individuals}}$ of all parasite pairs (with replacement)—necessarily correspond to stranger edges. Of the remaining proportion of pairwise comparisons, $m_{\mathrm{subgraph}}/n_{\mathrm{individuals}}$, the weighting of sibling/clonal edges is contingent on the underlying uniform distribution over transitive graphs of size $m_{\mathrm{subgraph}}$; the case $m_{\mathrm{subgraph}}=4$ is shown in Fig. B2a.

Population-level relationship graphs enriched for sibling and clonal edges naturally yield rapid growth in relatedness over successive generations of inbreeding. For parasite pairs connected by a stranger edge in generation *k*, parents are sampled independently from generation (k−1). Half-sibling, sibling, or clonal relationships between individuals connected by a stranger edge in generation *k* can therefore arise due to random sampling from the finite population in generation (k−1), and are a direct function of clonal structure in generation (k−1). However, we would expect half-sibling, sibling, and clonal relationships in generation *k* to be driven primarily by sibling/clonal edges within the population-level relatedness graph for generation *k* itself—particularly in early generations with limited clonal substructure.

Under our framework, we recover a mixture distribution for pairwise IBD sharing $R(j_{1},j_{2},k)$ in generation *k*—with a dominant component attributable to parasites separated by at least two generations and smaller components attributable to clones, siblings and half-siblings. The size $m_{\mathrm{subgraph}}$ of uniformly sampled transitive graphs with sibling/clonal/stranger edges (Taylor, Watson, *et al.* 2019; Taylor 2022b), relative to the parasite population size $n_{\mathrm{individuals}}$, governs the relative weightings of the half-sibling, sibling and clonal components to the mixture distribution—and consequently, the rate at which relatedness grows over successive generations.

##### B.5.3 Breeding between closely related parents: $p_{\mathrm{cotransmission}}$ vs $m_{\mathrm{subgraph}}$

For each sibling/clonal component within the population-graph of generation *k*, with probability $p_{\mathrm{cotransmission}}$, we have sampled parents without replacement from a subgraph of size $m_{\mathrm{subgraph}}$ in the population graph of generation (k−1). Under our simulation structure, each subgraph is itself sampled uniformly at random over the set of transitive relationship graphs of size $m_{\mathrm{subgraph}}$. The probability of a stranger–stranger cross vs a sibling–sibling cross vs selfing under this regime is therefore a function of $m_{\mathrm{subgraph}}$, with the case $m_{\mathrm{subgraph}}=4$ illustrated in Fig. B2b. The underlying rationale of this sampling scheme is to yield an enriched probability of sibling–sibling crosses, as a proxy for serial cotransmission (Wong *et al.* 2018); sampling from the constituent subgraphs *without* replacement augments the contribution of sibling–sibling crosses relative to selfing.

Setting $p_{\mathrm{cotransmission}}=0$—whereby the sampling of parents is predicated on stochastic drift only (Appendix B.5.2)—yields a “blindspot” in our simulated distributions of realized relatedness: in contrast to empirical estimates, we recover negligibly few *nonclonal* parasite pairs with IBD sharing exceeding 60%, signifying crosses between related parents. To recapitulate the smattering of parasite pairs with IBD sharing in the range 60%–90% seen in empirical estimates, we must inflate the probability of breeding between closely related parental lineages, consistent with evidence of extensive cotransmission (Nkhoma *et al*. 2020). We modulate $p_{\mathrm{contransmission}}>0$ and exploit substructure within simulated population-level relatedness graphs to mitigate this blindspot. While we have not explicitly embedded a mechanistic transmission model within our framework, subgraphs derived from parental lineages sampled from the population at large can be thought to encompass superinfection; while subgraphs derived from parental lineages sampled from a previous subgraph can be thought to correspond to cotransmission.

We note, however, that the augmented probability of sibling–sibling crosses under $p_{\mathrm{cotransmission}}>0$ yields elevated IBD sharing (in the vicinity of 60%–90%) for a relatively small proportion of parasite pairs: it primarily affects within-component comparisons for each sibling/clonal component in the accompanying population-level relationship graph, with an upper bound $m_{\mathrm{subgraph}}$ on the size of each sibling/clonal component. Between-cluster comparisons are largely modulated by stochastic drift, as discussed in Appendix B.5.2. Reverting to the mixture distribution interpretation of simulated pairwise IBD delineated in Appendix B.5.2, setting $p_{\mathrm{contransmission}}>0$ principally has the effect of broadening the sibling component, to include a small proportion of pairs reflecting breeding between recently related parents.

##### B.5.4 Linkage structure and spanning (n)IBD segments: $n_{\mathrm{markers}}$ vs $\bar{M}$

The mean length of consecutive markers $\bar{M}$ inherited from the same parent in a single meiosis governs the degree of dependence between loci, and consequently, the amount of linkage structure exhibited by related parasite pairs. For this linkage structure to emerge, however, the $n_{\mathrm{markers}}$ of interest must span sufficiently many segments of (n)IBD markers; and each segment of (n)IBD markers must be sufficiently long. As a function of the pairwise relatedness parameter $r^{\left(k,\ell \right)}$, the interplay between $\bar{M}$ and $n_{\mathrm{markers}}$ also governs the variance of the realized fraction of (polymorphic) sites IBD—which we treat as our truth value to diagnose biases in relatedness estimation.

We adopt a physical argument to recover $\bar{M}$ as a function of the marker count $n_{\mathrm{markers}}$: for some number of equidistant markers $n_{\mathrm{markers}}$, distributed across a chromosome of length *L* Morgans, the mean length of consecutive markers inherited from the same parent in a single meiosis is given by

$$\bar{M}(n_{\mathrm{markers}})=\frac{2}{1-e^{-L/n_{\mathrm{markers}}}},$$

obtained by plugging the inter-marker distance $L/n_{\mathrm{markers}}$ into Equation (B1).

We can derive a related metric for the average length of contiguous (n)IBD markers for independent crosses of a parental pair. For nonmeiotic siblings, loci ℓ and ℓ+1 (with intermarker distance $L/n_{\mathrm{markers}}$) share the same (n)IBD status with probability

P(dℓ+1=dℓ)=[0.5(1+e−L/nmarkers)]2⏟withineachnonmeioticsibling,lociℓandℓ+1inheritedfromthesameparent+[0.5(1−e−L/nmarkers)]2⏟withineachnonmeioticsibling,lociℓandℓ+1inheritedfromdifferentparents=0.5[1+e−2L/nmarkers].

For equidistant markers, the length of a contiguous segment of (n)IBD markers for nonmeiotic siblings is therefore geometrically distributed, with mean

$$\bar{Q}(n_{\mathrm{markers}})=\frac{2}{1-e^{-2L/n_{\mathrm{markers}}}}.$$

##### B.5.5 Minor allele frequency spectra: $p_{i}$

At each locus $i=1,\ldots ,n_{\mathrm{markers}}$, the count $n_{\mathrm{founders}}\cdot \theta_{\mathrm{founder}}(q,i)$ of each allele q∈{1,…,y} in the founder population (size $n_{\mathrm{founders}}$) follows a multinomial distribution

$$n_{\mathrm{founders}}\cdot \left(\theta_{\mathrm{founder}}(1,i),\ldots ,\theta_{\mathrm{founder}}(y,i)\right)∼\text{Multinomial}\left(n_{\mathrm{founders}},(p_{i}(1),\ldots ,p_{i}(y))\right)$$

where $p_{i}(q)$ is the probability of assigning allele *q* to a founder at locus *i*.

In this manuscript, we consider only biallelic loci, i.e. we fix y=2. The parameter $p_{i}$ governing founder sample allele frequencies $\theta_{\mathrm{founder}}$ has a strong bearing on minor allele frequency (MAF) spectra observed across successive generations. In the infinite generation limit, we would expect an allele $q_{i}\in \{1,2\}$ to reach fixation at each locus *i*, akin to the canonical Wright–Fisher model of stochastic drift (Etheridge 2011). As in the Wright–Fisher model, however, it is not necessarily the case that the *major* allele in the founder population reaches fixation. As such, in a transient setting, we can either observe allele frequencies becoming increasingly balanced over successive generations (as the minor allele grows in frequency); or increasingly unbalanced (as the major allele grows in frequency). For the most part, however, we expect a reduction in genetic diversity as crosses are repeatedly assigned major alleles.

To recover positively skewed biallelic MAF spectra with zero mode in the order of 10 generations, we set $\min\{p_{i}(1),p_{i}(2)\}\approx 0.1$, translating to unbalanced founder allele frequencies. Under this setting, however, we find that allele frequencies become increasingly balanced over successive generations for a subset of markers.

### Appendix C: Glossary of terms

$\bar{IBD}$ proportion of pairs of sampled parasites (with replacement, i.e. including self–self comparisons) that are IBD at a given locus.

$\bar{IBS}$ proportion of pairs of sampled parasites (with replacement, i.e. including self–self comparisons) that are IBS at a given locus.

**(n)IBD-to-observation model** inclusive of nIBD-to-allele, IBD-to-allele and nIBD-to-IBS models, under the assumed absence of genotyping error (whereby IBD necessarily implies IBS, i.e. IBD-to-IBS=1).

**background relatedness** under the simulation model, relatedness structure in generation zero parasites.

**genotype** a specific realization of the genome, which is a random variable distributed according to some ancestral process; in this study, we consider the genotype to be a sequence over all polymorphisms in the genome (elsewhere, its definition extends to subsets).

**inbreeding** recombination between genetically different but related parasites.

**individual** a parasite genotype.

**meiotic siblings** siblings derived from the same oocyst, i.e. siblings that are complements of one another and thus not independent.

**outbreeding** recombination between genetically unrelated parasites.

**realised relatedness** the fraction of polymorphic markers within the sample that are IBD for a given parasite pair.

**recent relatedness** under the simulation model, relatedness structure resulting from inbreeding from generation one onwards.

**relatedness structure** the locuswise partition of individuals in a parasite population or sample into transitive IBD clusters.

**sample** a collection of individuals, i.e. a collection of parasite genotypes.

**selfing** recombination between genetically identical parasites.

**strangers** a pair of individuals separated by two or more generations of recombination.
