# Supplementary material for: Systematic bias in malaria parasite relatedness estimation
Source: G3 (Bethesda). 2025 Jan 30;15(5):jkaf018. doi: 10.1093/g3journal/jkaf018 (PMC12060250; doi:10.1093/g3journal/jkaf018)
Supplement: jkaf018_Supplementary_Data [file jkaf018_supplementary_data.pdf]

ID	Accession	Country	Year
G1P001	SRR28619388	Guyana	2020
G1P002	SRR28619066	Guyana	2020
G1P005	SRR28619502	Guyana	2020
G1P007	SRR28619394	Guyana	2020
G1P009	SRR28619242	Guyana	2020
G1P010	SRR28619243	Guyana	2020
G1P013	SRR28619222	Guyana	2020
G1P015	SRR28619691	Guyana	2020
G1P024	SRR28619889	Guyana	2020
G1P025	SRR28619477	Guyana	2020
G1P026	SRR28619947	Guyana	2020
G1P028	SRR28619736	Guyana	2020
G1P029	SRR28619936	Guyana	2020
G1P030	SRR28619194	Guyana	
G1P031	SRR28619422	Guyana	2020
G1P032	SRR28619684	Guyana	2020
G1P033	SRR28619177	Guyana	2020
G1P034	SRR28619150	Guyana	
G1P035	SRR28619433	Guyana	2020
G1P036	SRR28619450	Guyana	
G1P051	SRR28619125	Guyana	2020
G1P073	SRR28619136	Guyana	2020
G1P076	SRR28619216	Guyana	2020
G1P080	SRR28619513	Guyana	2020
G1P091	SRR28619244	Guyana	2020
G1P100	SRR28619901	Guyana	2020
G1P101	SRR28619662	Guyana	2020
G1P105	SRR28619466	Guyana	2020
G1P111	SRR28619900	Guyana	2020
G1P112	SRR28619964	Guyana	2020
G1P115	SRR28619862	Guyana	2020
G1P120	SRR28619161	Guyana	2020
G1P156	SRR28619265	Guyana	2020
G1P157	SRR28619188	Guyana	2020
G1P158	SRR28619233	Guyana	2020
G1P160	SRR28619003	Guyana	2020
G1P161	SRR28619377	Guyana	2020
G1P162	SRR28619405	Guyana	2020
G1P166	SRR28619189	Guyana	2020
G1P167	SRR28619149	Guyana	2020

G1P168	SRR28619930	Guyana	2020
G1P170	SRR28619747	Guyana	2020
G1P171	SRR28619975	Guyana	2020
G1P172	SRR28619730	Guyana	2020
G1P173	SRR28619702	Guyana	2020
G1P175	SRR28619719	Guyana	2020
G4G007	SRR28619221	Guyana	2020
G4G008	SRR28619471	Guyana	2020
G4G010	SRR28619270	Guyana	2020
G4G013	SRR28619470	Guyana	2020
G4G015	SRR28619452	Guyana	2020
G4G016	SRR28619445	Guyana	2020
G4G024	SRR28619230	Guyana	2020
G4G025	SRR28619473	Guyana	2020
G4G026	SRR28619228	Guyana	2020
G4G027	SRR28619211	Guyana	2020
G4G028	SRR28619202	Guyana	2020
G4G035	SRR28619485	Guyana	2020
G4G036	SRR28619002	Guyana	2020
G4G041	SRR28619227	Guyana	2020
G4G043	SRR28619469	Guyana	2020
G4G044	SRR28619234	Guyana	2020
G4G045	SRR28619459	Guyana	2020
G4G047	SRR28619572	Guyana	2020
G4G048	SRR28619217	Guyana	2020
G4G049	SRR28619229	Guyana	2020
G4G050	SRR28619261	Guyana	2020
G4G055	SRR28619510	Guyana	2020
G4G057	SRR28619484	Guyana	2020
G4G060	SRR28619025	Guyana	2020
G4G061	SRR28619440	Guyana	2020
G4G063	SRR28619259	Guyana	2020
G4G064	SRR28619264	Guyana	2020
G4G067	SRR28619353	Guyana	2020
G4G069	SRR28619215	Guyana	2020
G4G070	SRR28619271	Guyana	
G4G073	SRR28619500	Guyana	2020
G4G074	SRR28619494	Guyana	2020
G4G089	SRR28619446	Guyana	2020
G4G093	SRR28619218	Guyana	2020
G4G094	SRR28619988	Guyana	2020

G4G096	SRR28619065	Guyana	2020
G4G098	SRR28619501	Guyana	2020
G4G100	SRR28619237	Guyana	2020
G4G103	SRR28619465	Guyana	2020
G4G105	SRR28619299	Guyana	2020
G4G114	SRR28619257	Guyana	2020
G4G118	SRR28619034	Guyana	2020
G4G128	SRR28619503	Guyana	2020
G4G130	SRR28619517	Guyana	2020
G4G131	SRR28619054	Guyana	2020
G4G134	SRR28619214	Guyana	2020
G4G138	SRR28619208	Guyana	2020
G4G141	SRR28619441	Guyana	2020
G4G154	SRR28619245	Guyana	2020
G4G156	SRR28619250	Guyana	2020
G4G157	SRR28619486	Guyana	2020
G4G161	SRR28619563	Guyana	2020
G4G162	SRR28619260	Guyana	2020
G4G164	SRR28619262	Guyana	2020
G4G167	SRR28619249	Guyana	2020
G4G172	SRR28619206	Guyana	2020
G4G174	SRR28619236	Guyana	2020
G4G177	SRR28619515	Guyana	2020
G4G179	SRR28619458	Guyana	2020
G4G180	SRR28619210	Guyana	2020
G4G182	SRR28619112	Guyana	2020
G4G185	SRR28619480	Guyana	2020
G4G186	SRR28619253	Guyana	2020
G4G190	SRR28619487	Guyana	2020
G4G191	SRR28619781	Guyana	2020
G4G192	SRR28619258	Guyana	2020
G4G193	SRR28619765	Guyana	2020
G4G195	SRR28619464	Guyana	2020
G4G198	SRR28619467	Guyana	2020
G4G199	SRR28619203	Guyana	2020
G4G201	SRR28618995	Guyana	2020
G4G202	SRR28619364	Guyana	2020
G4G205	SRR28619479	Guyana	2020
G4G209	SRR28619266	Guyana	2020
G4G213	SRR28619451	Guyana	2020
G4G215	SRR28619643	Guyana	2020

G4G223	SRR28619231	Guyana	2020
G4G225	SRR28619850	Guyana	2020
G4G237	SRR28619476	Guyana	2020
G4G239	SRR28619200	Guyana	2020
G4G241	SRR28619198	Guyana	2020
G4G242	SRR28619630	Guyana	2020
G4G246	SRR28619453	Guyana	2020
G4G253	SRR28619483	Guyana	2020
G4G255	SRR28619834	Guyana	2020
G4G256	SRR28619220	Guyana	2020
G4G257	SRR28619444	Guyana	2020
G4G258	SRR28620549	Guyana	2020
G4G263	SRR28620550	Guyana	2020
G4G267	SRR28619753	Guyana	2020
G4G270	SRR28619255	Guyana	2020
G4G271	SRR28619552	Guyana	2020
G4G274	SRR28619196	Guyana	2020
G4G278	SRR28619014	Guyana	2020
G4G281	SRR28619241	Guyana	2020
G4G282	SRR28619447	Guyana	2020
G4G284	SRR28619815	Guyana	2020
G4G288	SRR28619276	Guyana	2020
G4G305	SRR28619213	Guyana	2020
G4G306	SRR28619457	Guyana	2020
G4G318	SRR28619207	Guyana	2020
G4G323	SRR28619474	Guyana	2020
G4G324	SRR28619481	Guyana	2020
G4G327	SRR28619770	Guyana	2020
G4G330	SRR28619882	Guyana	2020
G4G331	SRR28619654	Guyana	2020
G4G339	SRR28619463	Guyana	2020
G4G342	SRR28619526	Guyana	2020
G4G346	SRR28619197	Guyana	2020
G4G351	SRR28619235	Guyana	2020
G4G353	SRR28619204	Guyana	2020
G4G356	SRR28619871	Guyana	2020
G4G357	SRR28619454	Guyana	2020
G4G359	SRR28619343	Guyana	2020
G4G360	SRR28619478	Guyana	2020
G4G362	SRR28619332	Guyana	2020
G4G371	SRR28619267	Guyana	2020

G4G372	SRR28619537	Guyana	2020
G4G373	SRR28619195	Guyana	2020
G4G380	SRR28619506	Guyana	2020
G4G384	SRR28619448	Guyana	2020
G4G392	SRR28619752	Guyana	2020
G4G396	SRR28619256	Guyana	2020
G4G404	SRR28620906	Guyana	2020
G4G408	SRR28620911	Guyana	2020
G4G409	SRR28620910	Guyana	2020
G4G410	SRR28620909	Guyana	2020
G4G411	SRR28620579	Guyana	2020
G4G414	SRR28619263	Guyana	2020
G4G422	SRR28620575	Guyana	2020
G4G427	SRR28620576	Guyana	2020
G4G429	SRR28620577	Guyana	2020
G4G434	SRR28620892	Guyana	2020
G4G439	SRR28620893	Guyana	2020
G4G443	SRR28620894	Guyana	2020
G4G444	SRR28619247	Guyana	2020
G4G446	SRR28620896	Guyana	2020
G4G447	SRR28620895	Guyana	2020
G4G450	SRR28620898	Guyana	2020
G4G452	SRR28620899	Guyana	2020
G4G463	SRR28620902	Guyana	2020
G4G469	SRR28620904	Guyana	2020
G7B002	SRR28621086	Guyana	2020
G7B003	SRR28621087	Guyana	2020
G7B005	SRR28621090	Guyana	2020
G7B008	SRR28621091	Guyana	2020
G7B017	SRR28621092	Guyana	2020
G7B018	SRR28621093	Guyana	2020
G7B021	SRR28621094	Guyana	2020
G7B028	SRR28620559	Guyana	2020
G7B035	SRR28621069	Guyana	2020
G7B037	SRR28621070	Guyana	2020
G7B041	SRR28621071	Guyana	2020
G7B043	SRR28621072	Guyana	2020
G7B051	SRR28621073	Guyana	2020
G7B054	SRR28621074	Guyana	2020
G7B056	SRR28621075	Guyana	2020
G7B057	SRR28621076	Guyana	2020

G7B066	SRR28621082	Guyana	2020
G7B069	SRR28621083	Guyana	2020
G7B071	SRR28621084	Guyana	2020
G7B072	SRR28619750	Guyana	2020
G7B073	SRR28621085	Guyana	2020
G7B074	SRR28620553	Guyana	2020
G7B081	SRR28620554	Guyana	2020
G7B082	SRR28620555	Guyana	2020
G7B084	SRR28620556	Guyana	2020
G7B088	SRR28619749	Guyana	2020
G7B095	SRR28620558	Guyana	2020
G7B098	SRR28621095	Guyana	2020
G7B100	SRR28621096	Guyana	2020
G7B101	SRR28621097	Guyana	2020
G7B107	SRR28621098	Guyana	2020
G7B108	SRR28621099	Guyana	2020
G7B109	SRR28621101	Guyana	2020
G7B117	SRR28620560	Guyana	2020
G7B127	SRR28620562	Guyana	2020
G7B130	SRR28619746	Guyana	2020
G7B139	SRR28619745	Guyana	2020
G7B140	SRR28620532	Guyana	2020
G7B142	SRR28620533	Guyana	2020
G7B145	SRR28620534	Guyana	2020
G7B146	SRR28620535	Guyana	2020
G7B147	SRR28620536	Guyana	2020
G7B148	SRR28620537	Guyana	2020
G7B149	SRR28620538	Guyana	2020
G7B151	SRR28620539	Guyana	2020
G7B155	SRR28620540	Guyana	2020
G7B164	SRR28620543	Guyana	2020
G7B165	SRR28620544	Guyana	2020
G7B167	SRR28620545	Guyana	2020
G7B174	SRR28620548	Guyana	2020
G7B178	SRR28620551	Guyana	2020
G7B187	SRR28620568	Guyana	2020
G7B189	SRR28620569	Guyana	2020
G7B193	SRR28619748	Guyana	2020
G7B194	SRR28620572	Guyana	2020
G7B195	SRR28620571	Guyana	2020
G7B197	SRR28620573	Guyana	2020

G7B199	SRR28621080	Guyana	2020
G7B210	SRR28620564	Guyana	2020
G7B217	SRR28620566	Guyana	2020
G7B225	SRR28620567	Guyana	2020
GUY0123	SRR28619739	Guyana	2017
GUY0154	SRR28619186	Guyana	2017
GUY0159	SRR28619961	Guyana	2017
GUY0166	SRR28619971	Guyana	2017
GUY0174	SRR28619982	Guyana	2017
T104	SRR28619951	Guyana	2016
T118	SRR28619957	Guyana	2016
T133	SRR28619941	Guyana	2016
T145_swga	SRR11103254	Guyana	2016
T158	SRR28619404	Guyana	2016
T159	SRR28619401	Guyana	2016
T211	SRR28619687	Guyana	2016
T215	SRR28619378	Guyana	2016
T227	SRR28619397	Guyana	2016
T230	SRR28619698	Guyana	2016
T240	SRR28619146	Guyana	2016
T285	SRR28619383	Guyana	2016
T301	SRR28619381	Guyana	2016
T359	SRR28619890	Guyana	2016
T373	SRR28619647	Guyana	2016
T377	SRR28619902	Guyana	2016
T387	SRR28619677	Guyana	2016
T392	SRR28619899	Guyana	2016
T482	SRR28619846	Guyana	2016
T497	SRR28619365	Guyana	2016
T640	SRR28619609	Guyana	2016
T679	SRR28619836	Guyana	2016
T824	SRR28619801	Guyana	2016
T827	SRR28619303	Guyana	2016
PW0069-C	ERR1818180	Colombia	2014-17
PW0070-C	ERR1818181	Colombia	2014-17
PW0008-C	ERR039903	Colombia	1993-2007
PW0002-C	ERR039930	Colombia	1993-2007
PW0009-C	ERR039986	Colombia	1993-2007
PW0016-C	ERR039988	Colombia	1993-2007
PW0007-C	ERR042222	Colombia	1993-2007
PW0017-C	ERR042223	Colombia	1993-2007

PW0012-C	ERR042224	Colombia	1993-2007
PW0004-C	ERR042226	Colombia	1993-2007
PW0003-C	ERR042227	Colombia	1993-2007
PW0001-C	ERR042228	Colombia	1993-2007
PW0005-C	ERR042229	Colombia	1993-2007
PW0006-C	ERR042230	Colombia	1993-2007
PW0013-C	ERR042231	Colombia	1993-2007
PW0015-C	ERR042232	Colombia	1993-2007
PW0014-C	ERR042233	Colombia	1993-2007
PW0053-C	ERR1818164	Colombia	2014-17
PW0057-C	ERR1818168	Colombia	2014-17
PW0067-C	ERR1818178	Colombia	2014-17
PW0105-C	ERR1911267	Colombia	2014-17
SPT26227	ERR2496598	Colombia	2014-17
PW0073-C	ERR1818184	Colombia	2014-17
PW0088-C	ERR1911250	Colombia	2014-17
SPT26335	ERR2496593	Colombia	2014-17
SPT26336	ERR2496568	Colombia	2014-17
SPT26229	ERR2496572	Colombia	2014-17
SPT26248	ERR2496548	Colombia	2014-17
